# Procedural and Methodological Quality in Preclinical Stroke Research–A Cohort Analysis of the Rat MCAO Model Comparing Periods Before and After the Publication of STAIR/ARRIVE

**DOI:** 10.3389/fneur.2022.834003

**Published:** 2022-05-30

**Authors:** Jacqueline Friedrich, Ute Lindauer, Anke Höllig

**Affiliations:** Department of Neurosurgery, University Hospital RWTH Aachen, Aachen, Germany

**Keywords:** MCAO, rat stroke model, preclinical stroke research, CBF monitoring, experimental quality assurance, STAIR, ARRIVE

## Abstract

The translation of preclinical stroke research into successful human clinical trials remains a challenging task. The first Stroke Therapy Academic Industry Roundtable (STAIR) recommendations for preclinical research and several other guidelines were published to address these challenges. Most guidelines recommend the use of physiological monitoring to detect the occurrence of undesired pathologies such as subarachnoid hemorrhage and to limit the variability of the infarct volume and–therefore-homogenize the experimental result for complete reporting particularly with respect to transparency and methodological rigor. From the years 2009 and 2019, 100 published articles each using a rat stroke model were analyzed to quantify parameters related to anesthesia, physiological monitoring, stroke model type, ischemia verification, and overall study quality over time. No significant difference in the frequency of cerebral blood flow (CBF) measurements over time (28/34% for 2009/2019) was found. Notably, significantly fewer studies reported temperature, blood pressure, and blood gas monitoring data in 2019 compared to 2009. On the other hand, an increase in general study quality parameters (e.g., randomization, reporting of approval) was seen. In conclusion, the frequency of periinterventional monitoring has decreased over time. Some general methodological quality aspects, however, partially have increased. CBF measurement–the gold standard for ischemia verification-was applied rarely. Despite the growing recognition of current guidelines such as STAIR and ARRIVE (both widely approved in 2019) reporting, methods and procedures mostly do not follow these guidelines. These deficits may contribute to the translational failure of preclinical stroke research in search for neuroprotective therapies.

## Introduction

Stroke is the second most common cause of death and adult disability worldwide (1). Globally, 5.5 million people of all ages and both sexes die from stroke annually (2). According to the 2019 Global Burden of Disease Study, stroke remains the second leading cause of global disability-adjusted life-years (DALYs) in patients over age 50 since 1990 (3). Even though there has been a substantial decline in stroke age-standardized DALY rates since 1990 (3), the development of safe and effective treatments is still a major challenge for experimental and clinical neuroscience.

Preclinical stroke research has helped a lot toward a deeper pathophysiological understanding of stroke (4). Further, due to complexity of the disease including multiple interactions (between different organs such as brain–heart interactions (5)) as well as influence of various systems such as the immune system, which shows a pronounced reaction after ischemic stroke (6) preclinical stroke research constitutes an important pillar of stroke research. However, many experimental stroke treatments with regard to neuroprotective agents result in reduction of infarct size and improved clinical presentation in animal models, most ultimately fail when translated into clinical trials (7). As a consequence, both traditional animal models *per se* and the documentation/reporting of preclinical stroke studies have to be reviewed.

From a methodological point of view, during the last decades several reforms mostly in terms of guidelines have been made: Probably the best known guideline in this field is the Stroke Therapy Academic Industry Roundtable (STAIR) guideline published in 1999 (8) and updated in 2009 (9). In the following years, numerous other guidelines for preclinical stroke trials were presented (10–13). Further, the ARRIVE criteria (Animal Research: Reporting of *in vivo* Experiments) depicting general recommendations to improve the reporting of research involving animals were published in 2010 and updated in 2020 (14, 15). Besides, some journals such as *Stroke* provide a Basic Science Checklist requesting details on methodological quality such as on the randomization and blinding procedures, definition of inclusion and exclusion criteria etc., which may increase transparent reporting (16).

Undoubtedly, there are numerous potential reasons for translational failure of preclinical stroke research (particularly with respect to neuroprotectants) conditioned by the experimental setting itself, such as: Generation of a plausible hypothesis, methodological quality of study planning, adequate performance and surveillance of the experimental procedure, objective (ideally blinded) analyses of study results, and full and transparent reporting.

Thus, the experimental procedure itself or rather the appropriateness of its performance may contribute to translational success or failure. With respect to the most common experimental setting in preclinical stroke research in rats, the middle cerebral artery occlusion (MCAO), previous publications have highlighted the need for an appropriate ischemia verification using methods such as cerebral blood flow (CBF) measurement (17–22). Model-immanent confounders and complications (such as inadvertent induction of subarachnoid hemorrhage–SAH-or insufficient MCAO) may not be prevented by additional monitoring, however, usage of tools like CBF measurement may allow an instantaneous detection of the experimental result including the occurrence of undesired pathologies such as SAH. This is essential because applying the MCAO model, induction of SAH instead or in parallel to ischemia is a common phenomenon (comprising up to 30% of the experiments) (17). Thus, results may be biased by an inadequate modeling of the initially aspired pathology. As Philip and colleagues pointed out, “…the reliability of the model to induce ischemia and reproducibly cause infarction…” is hampered by a lack of CBF monitoring (23). Therefore, it is essential to question also the performance of disease models in order to allow an adequate interpretation and classification of the results.

Thus, the aim of the study was to document the performance of the rat MCAO procedure over time representing the most commonly used stroke model besides the murine MCAO procedure. Considering the 2009 STAIR update as well as the publication of the ARRIVE criteria in 2010, we compared the years 2009 vs. 2019 (analyzing a sample of 100 original articles each year) with particular respect to periinterventional monitoring and methods of ischemia verification (focusing on CBF measurement). Further, aspects of methodological quality (such as sample size calculation) were evaluated over time (before and after the public awareness of STAIR and ARRIVE). Thus, the results will provide an overview of methodological and periinterventional/procedural quality control of studies applying the intraluminal rat stroke model over time. An additional quality score to assess methodological and procedural aspects is provided. Hence, conclusions may be drawn regarding the relevance of the experimental results and failure of translation may be detected due to inaccurate modeling or inappropriate methodology.

## Materials and Methods

This review followed the STROBE guidelines (Strengthening the Reporting of Observational Studies in Epidemiology) (24). Our study is exempt from ethics approval because we collected and processed data from previous animal studies in which ethics approval has already been obtained.

### Search Strategy

Literature research was conducted on 09 August 2020 on MEDLINE database via PubMed using the search strategy: *((((tMCAO) OR (transient middle cerebral artery occlusion)) OR (middle cerebral artery occlusion)) OR (MCAO)) AND (rat)*. A time filter was applied to the search results to select only publications from 2009 and 2019, and the results for each year were sorted in ascending order according to their publication date. The results of both years were screened with regard to our inclusion criteria. Only articles that met the following criteria were included:

- Written in English.
- Original research article.
- Rat model.
- Occlusion of MCA.
- Intraluminal thread model.

Screening was continued until 100 articles for each year could be included.

### Data Extraction

The first author (JF) extracted the data, which was validated by the last author (AH). Data on the parameters listed in **Table 2** were taken from each study. If no information was available on a parameter in the article or its supplementary material, it was also documented as “not reported.” The parameter concerned was also documented as “not reported” if only a reference was provided without further information. Laser Doppler measurement was considered standard measurement. Other methods that could be potentially suitable for ischemia verification were only recorded when each animal included in the study was subjected to at least one of them. In case only a proportion was analyzed the variable “other potential suitable methods” was assessed as “none.” The factor “a priori sample size calculation” was recorded as “not applicable” in case of an exploratory study. If a study does not include different groups “randomization” was assessed as “not applicable.” Neurological assessments were only considered as a potentially suitable method for ischemia verification if no treatment had been performed previously (as usually treatment is supposed to alter/improve neurostatus, thus, treatment may cover the induced neurological deficit).

Subsequently, the impact factor of the journal in which the article was published was determined for the year of publication via Web of Science.

### Analysis

We defined a quality score that includes information on anesthesia monitoring, ischemia verification, and general quality criteria (Table 1) to analyze study quality. Category 1 comprises five items with a maximum score of 5. The parameter “ventilation” was not included due to the guidance that “unnecessary use of mechanical ventilation should be avoided when a particular MCAO model is not likely to cause respiratory problems. Ventilation may be needed when the operation lasts long (>1 h) and when the ischemia affects brain stem function” (11). We did not include ventilation in the assessment of study quality as it is possible that the experimenters deliberately chose not to ventilate in accordance with this recommendation. Category 2 comprises two items with a maximum score of 3. For the item “CBF measurement” the statements “not clearly reported” and “unilateral” were given equal scores as we assumed a unilateral measurement in most cases where the CBF measurement was ambiguously described. Category 3 includes four items with a maximum score of 4. Each study was assigned a score from 0 (lowest quality) to 12 (highest quality).

**Table 1:** Quality score items.

**Category**	**Item**	**Scoring**
Category 1: Anesthesia monitoring	Anesthesia	Not reported = 0 Yes = 1
	Temperature	Not reported = 0 Yes = 1
	Heart rate	Not reported = 0 Yes = 1
	Blood pressure	Not reported = 0 yes = 1
	Blood gases/ O_2_ saturation	Not reported = 0 Yes = 1
Category 2: Ischemia verification	CBF measurement	Not reported = 0 Not clearly reported/unilateral = 1 Bilateral = 2
	Other potential suitable methods	Not reported = 0 Yes = 1
Category 3: Quality standards	Approved license	Not reported = 0 Yes = 1
	A priori sample size calculation	Not reported = 0 Yes/not applicable = 1
	Randomization	Not reported = 0 Yes/not applicable = 1
	Inclusion/exclusion criteria	Not or not clearly reported = 0 Yes = 1

*CBF, cerebral blood flow*.

### Statistics

All statistical analyses were performed using Jamovi 1.6.15 (25) with the level of significance set at *p* < 0.05. For each parameter category the Fisher exact test was applied to check whether there were significant differences between 2009 and 2019. To compare the impact factors for 2009 and 2019 the Mann-Whitney test for non-parametric data was used. The quality score related to the continent of origin was analyzed using the Kruskal-Wallis test. The Spearman's correlation analysis was performed to evaluate associations between the quality score and the impact factor. Graphs were created using GraphPad Prism 9.1.1 (LaJolla, USA).

## Results

**Figure 1:**
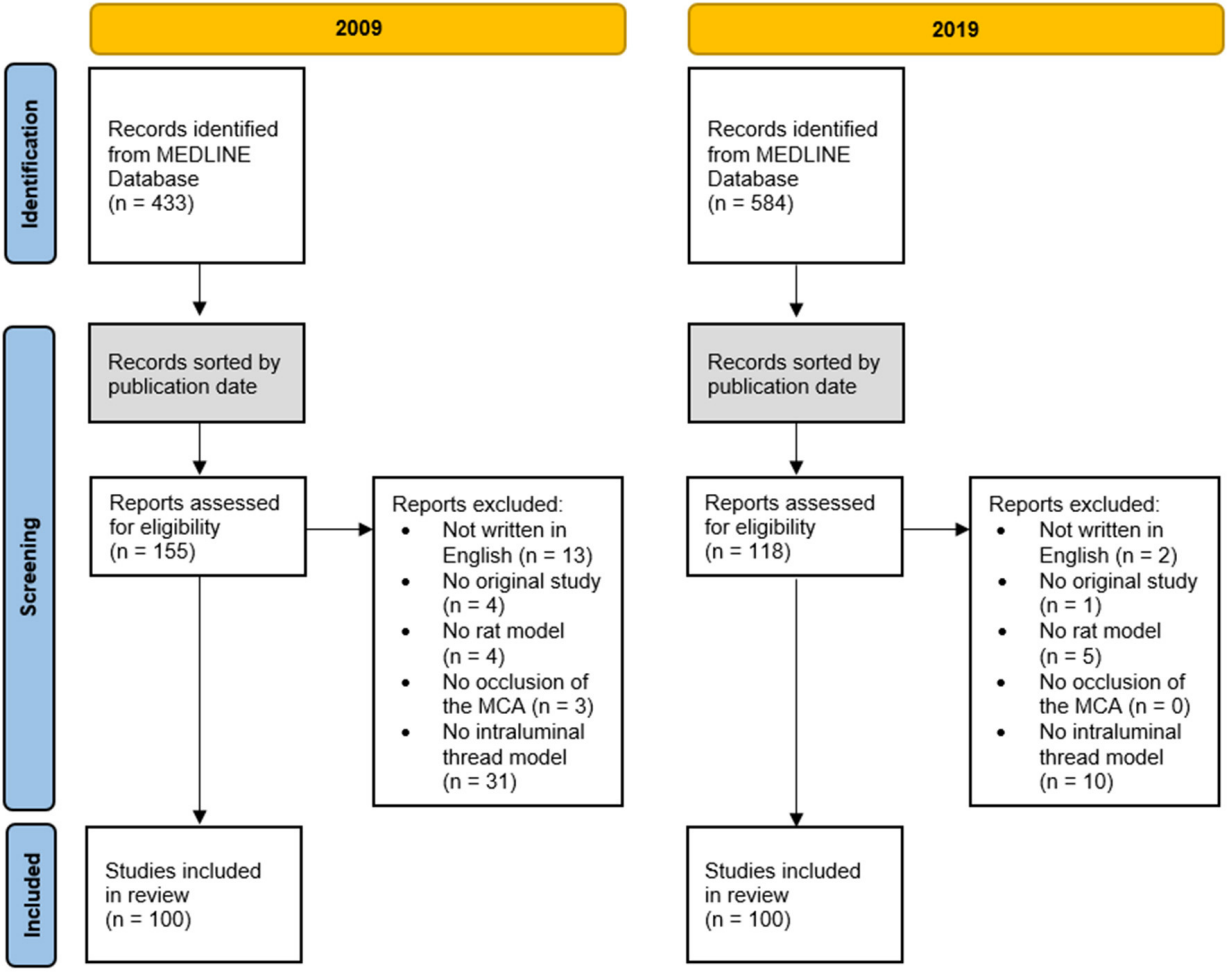
Flow of database search, screening, eligibility, selection, and inclusion of studies.

A PubMed search (search strategy: *((((tMCAO) OR (transient middle cerebral artery occlusion)) OR (middle cerebral artery occlusion)) OR (MCAO)) AND (rat))* revealed 433 hits for 2009 and 584 hits for 2019. The results were sorted in ascending order by their publication date and then subsequently screened for relevance and eligibility until a total of 100 studies for each year were available (a list of all studies included can be found in the Supplementary Material). The numbers of publications rejected and the reasons for exclusion are given in Figure 1. In 2009, a last author publishing two manuscripts was observed twice and publishing three manuscripts thrice. In 2019, in five cases a last author was found publishing two manuscripts. The results for each parameter analyzed from the 100 sample articles published in 2009 were compared with those published in 2019 and are presented hereinafter.

### Rat

Sprague Dawley was the most common strain in 2009 (65%) and 2019 (80%), with a significant increase in 2019 (*p* = 0.026). Both in 2009 and 2019, a total of 88.2% of the rats were male. In both years, the majority of animals had a mean weight of 250–300 g (52% in 2009, 51% in 2019). In the most cases, the weight range of the animals was relatively small.

### Anesthesia and Physiological Monitoring

In 2009, inhalation anesthesia was used in 51% of the studies, whereas in 38% injection anesthesia was used. In 2019, inhalation anesthesia was used significantly less often (33%, *p* = 0.015) and injection anesthesia was applied more frequently (57%, *p* = 0.011).

The majority of studies did not report the mode of ventilation (84% in 2009, 91% in 2019). Intubation was reported in 8 studies in 2009 and 2 in 2019 (8/2%). Mask ventilation was reported in 7 studies (7%) each 2009 and 2019.

Temperature control was documented significantly less frequently in 2019 compared with 2009 (74% in 2009, 52% in 2019, *p* = 0.002).

Further, the mention of heart rate monitoring was scarce at both time points (missing information in 93% in 2009, 96% in 2019). Both blood pressure monitoring (missing information in 78% in 2009, 94% in 2019, *p* = 0.002) and monitoring of blood gases and O_2_ saturation (missing information in 76% in 2009, 92% in 2019, *p* = 0.003) in general were rarely reported. In summary, the documentation of periinterventional physiological parameters was lower in 2019 compared to 2009 (for all parameters analyzed).

Details are summarized in Table 2.

**Table 2:** Basic experimental characteristics extracted from the studies included in the review.

	**Parameter**	**Category**		**Year**		***p*-value**
				**2009**	**2019**	
				***n* [%]^a^**		
Rat	Strain	Not reported		2	0	0.497
		Sprague Dawley		65	80	0.026^*^
		Wistar		29	17	0.064
		Other		4	1	0.369
		Mixed		0	2	0.497
	Sex^b^	Not reported		9 [8.8%]	5 [4.9%]	0.407
		Male		90 [88.2%]	90 [88.2%]	1.000
		Female		2 [1.7%]	7 [6.9%]	0.170
		Castrated male		1 [1.0%]	0 [0.0%]	1.000
	Weight	Mean	Not reported	7	13	0.238
			150–200 g	0	5	0.059
			>200–250 g	17	21	0.589
			>250–300 g	52	51	1.000
			>300–350 g	21	7	0.007^**^
			>350–400 g	3	2	1.000
			>400–450 g	0	1	1.000
		Variability	Not reported	7	13	0.238
			0 g	3	2	1.000
			>0–20 g	12	23	0.062
			>20–40 g	33	30	0.761
			>40–60 g	36	26	0.169
			>60–80 g	7	5	0.767
			>80–100 g	2	0	0.497
			>180–200 g	0	1	1.000
Anesthesia and physiological monitoring	Anesthesia	Not reported		11	10	1.000
		Inhalation		51	33	0.015^*^
		Injection		38	57	0.011^*^
	Ventilation	Not reported		84	91	0.199
		Intubation		8	2	0.101
		Mask		7	7	1.000
		Tracheostomy		1	0	1.000
	Temperature	Not reported		26	48	0.002^**^
		Yes		74	52	0.002^**^
	Heart rate	Not reported		93	96	0.537
		Yes		7	4	0.537
	Blood pressure	Not reported		78	94	0.002^**^
		Yes		22	6	0.002^**^
	Blood gases/O_2_ saturation	Not reported		76	92	0.003^**^
		Yes		24	8	0.003^**^
MCAO model	Reperfusion^b^	Not reported		3 [2.7%]	3 [2.9%]	1.000
		Yes		87 [79.1%]	85 [83.3%]	0.484
		No		20 [18.2%]	14 [13.7%]	0.455
	Occlusion duration^b^	Not reported		3 [2.7%]	4 [3.9%]	0.713
		Short transient (≤60 min)		22 [20.0%]	19 [18.6%]	0.863
		Long transient (>60 min)		65 [59.1%]	65 [63.7%]	0.573
		Permanent		20 [18.2%]	14 [13.7%]	0.455
	Filament type	Not reported		15	9	0.276
		Nylon uncoated		21	24	0.735
		Nylon poly-L-lysine coated		5	2	0.445
		Nylon silicone coated		20	30	0.141
		Nylon other coating		2	3	1.000
		PE-50 catheter		1	1	1.000
		Nylon uncoated + blunted tip		29	22	0.330
		Nylon poly-L-lysine coated + blunted tip		5	6	1.000
		Nylon silicone coated + blunted tip		1	3	0.621
		PE-50 catheter + blunted tip		1	0	1.000
Ischemia verification	Number of methods	0		27	16	0.084
		1		37	35	0.883
		2		28	38	0.176
		3		8	9	1.000
		4		0	1	1.000
		5		0	1	1.000
	CBF measurement	Not reported		72	66	0.445
		Measured region not clearly reported		15	25	0.111
		Unilateral		13	7	0.238
		Bilateral		0	2	0.497
	Other potential suitable methods^b^	None		33 [26.8%]	31 [21.8%]	0.389
		MRI		10 [8.1%]	12 [8.5%]	1.000
		Neurological assessment		19 [15.4%]	27 [19.0%]	0.516
		TTC staining		33 [26.8%]	38 [26.8%]	1.000
		Morphological staining^c^		25 [20.3%]	32 [22.5%]	0.765
		F-18 FDG PET/CT		0 [0.0%]	2 [1.4%]	0.501
		Cerebral tissue oxygen pressure (PtiO_2_)		1 [0.8%]	0 [0.0%]	0.464
		ICP monitoring		1 [0.8%]	0 [0.0%]	0.464
Sham group		No sham group		35	16	0.003^**^
		Not explicitly reported		33	47	0.060
		Surgery without filament insertion		23	30	0.336
		Surgery with filament insertion		8	5	0.568
		Without surgery		1	2	1.000

a *Frequencies in % are only given in case of n ≠100*.

b *Parameter with multiple mentions*.

c *Contains hematoxylin and eosin (H&E), Nissl, cresyl violet, Evans blue, Luxol Fast Blue, toluidine blue, pimonidazole and TUNEL staining*.

* *p ≤ 0.05*;

** *p ≤ 0.01. MCAO, middle cerebral artery occlusion; CBF, cerebral blood flow; TTC, 2,3,5-Triphenyltetrazolium chloride; F-18, fluorine-18; FDG, fluorodeoxyglucose; PET/CT, positron emission tomography/computed tomography; ICP, intracranial pressure*.

### MCAO Model

In 2009, 20.0% of studies chose a short transient occlusion duration of ≤60 min, compared to 18.6% in 2019. A long transient occlusion duration of >60 min was applied in 59.1% of cases in 2009 and in 63.7% in 2019. Permanent occlusion was performed in 18.2% of studies in 2009 and 13.7% in 2019.

The most commonly used filament types were uncoated nylon thread with blunted tip (29% in 2009, 22% in 2019), uncoated nylon filament (21% in 2009, 24% in 2019), and silicone coated nylon thread (20% in 2009, 30% in 2019).

In 35% (2009), respectively 16% (2019) no sham group was mentioned. If sham groups were documented, definitions for “Sham” varied between the studies (surgery without filament insertion, surgery with filament insertion, no surgery at all).

### Ischemia Verification

CBF measurement was reported poorly: In 2009, only 28% and in 2019 only 34% documented any type of CBF measurement. Out of the studies mentioning CBF measurement, the exact region of interest was not clearly described in 15% (2009) and 25% (2019). A unilateral measurement was performed in 13% of the studies in 2009 and in 7% of the studies in 2019. A bilateral measurement was documented in none of the articles analyzed for 2009 and in only 2% of cases in 2019.

Both in 2009 and 2019, the most commonly used “alternative” methods suitable for ischemia verification (besides CBF measurement) were 2,3,5-Triphenyltetrazolium chloride (TTC) staining (each 26.8%), morphological staining techniques (20.3% in 2009, 22.5% in 2019), neurological assessments (15.4% in 2009, 19.0% in 2019), and magnetic resonance imaging (MRI) (8.1% in 2009, 8.5% in 2019).

Of note, if functional testing was included into the measures for ischemia verification, still 27% in 2009 and 16% in 2019 did not perform any method of ischemia verification.

In the majority of publications, a sensorimotor score was used to assess neurological status (2009: 100%; 2019: 22.9%); complex behavioral testing was scarce (see Table 3). The most common tests were the 5-point scale (2009: 36.8%; 2019: 70.4%), 12-point scale (2009: 15.8%; 2019: 0%) and 18-point scale (2009: 5.3%; 2019: 11.1%).

**Table 3:** Neurological assessments extracted from the studies included in the review.

	**Parameter**	**Category**	**Year**		***p*-value**
			**2009**	**2019**	
			***n*** **[%]**		
Ischemia verification	Neurological assessment	Not clearly reported	0 [0.0%]	1 [3.7%]	1.000
		Tests for sensorimotor function	19 [100%]	24 [88.9%]	0.257
		Sensorimotor function tests + tests for cognition / memory function	0 [0.0%]	2 [7.4%]	0.504

### Quality Standards

In 2009, only 59 studies documented the approval by responsible animal welfare authorities, 56 of them did not specify a license number. In 2019, significantly more studies reported a study approval (85%, *p* < 0.001). Out of the approved studies, in 24% a license number was provided (vs. 3% in 2009; *p* < 0.001).

The majority of all studies did not report if an a priori sample size calculation was performed (99% in 2009, 92% in 2019, *p* = 0.035). Of note, both in the article samples from 2009 and 2019, a sample size calculation was presented in <10%.

Randomization was significantly more commonly applied in 2019 compared to 2009 (39% in 2009, 73% in 2019, *p* < 0.001).

A clear description of inclusion or exclusion criteria was often not reported, notably with a tendency toward poorer reporting in 2019 (49% in 2009, 32% in 2019, *p* = 0.021).

The results are summarized in Table 4.

**Table 4:** General quality standards extracted from the studies included in the review.

	**Parameter**	**Category**	**Year**		***p*-value**
			**2009**	**2019**	
			***n***		
Quality standards	Approved license	Not reported	41	15	<0.001^***^
		Yes, without license number	56	61	0.566
		Yes, with license number	3	24	<0.001^***^
	A priori sample size calculation	Not reported	99	92	0.035^*^
		Yes	1	7	0.065
		Not applicable	0	1	1.000
	Randomization	Not reported	61	24	<0.001^***^
		Yes	39	73	0.001^***^
		Not applicable	0	3	0.246
	Blinding for neurological assessment	Not reported	12	22	0.089
		Yes	7	5	0.767
		No/no suitable neurological assessment	81	73	0.239
	Inclusion/exclusion criteria	Not reported	49	32	0.021^*^
		Yes	35	47	0.114
		Not explicitly reported	16	21	0.467

* *p ≤ 0.05*,

*** *p ≤ 0.001*.

### Country/Continent of Origin

The studies analyzed were conducted in 20 different countries worldwide. In 2009, most studies were conducted in the USA (33%). In 2019, significantly fewer studies were conducted in the USA (13%, *p* = 0.001). By contrast, significantly more studies were conducted in China in 2019 (67%) than in 2009 (25%, *p* < 0.001) (please see Table 5).

**Table 5:** Number of studies conducted in a given country and impact factors of the studies included in the review.

			**Year**		***p*-value**
			**2009**	**2019**	
			***n***		
Country		Brazil	0	1	1.000
		Canada	1	0	1.000
		China	25	67	<0.001^***^
		Czech Republic	0	1	1.000
		Finland	1	0	1.000
		France	2	0	0.497
		Germany	3	2	1.000
		India	2	1	1.000
		Iran	2	2	1.000
		Italy	2	0	0.497
		Japan	11	3	0.049
		Netherlands	1	0	1.000
		Poland	1	1	1.000
		Republic of Korea	8	2	0.101
		Singapore	0	1	1.000
		Sweden	1	1	1.000
		Taiwan	2	3	1.000
		Turkey	2	1	1.000
		UK	3	1	0.621
		USA	33	13	0.001^***^
Continent		Asia	52	80	<0.001^***^
		Europe	14	6	0.097
		North America	34	13	<0.001^***^
		South America	0	1	1.000
Relevance	Impact factor	Mean	3.246	3.646	0.095
		SD	1.705	1.669	

*** *p ≤ 0.001*.

### Impact Factors

The mean impact factors of the articles did not vary significantly between 2009 and 2019 (*p* = 0.095). The impact factors of the journals correlated only weakly with the quality scores (see below) (2009: *r* = 0.158; *p* = 0.117; 2019: *r* = 0.235; *p* = 0.02).

### Quality Score

Out of the assessed parameters, a quality score system (consisting of items for “anesthesia monitoring”–category 1, “ischemia verification”-category 2 and “general quality standards”–category 3) was formed in order to mirror methodological and experimental quality standards. A maximum of 12 points could be achieved (category 1: 5 points; category 2: 3 points; category 3: 4 points).

**Figure 2:**
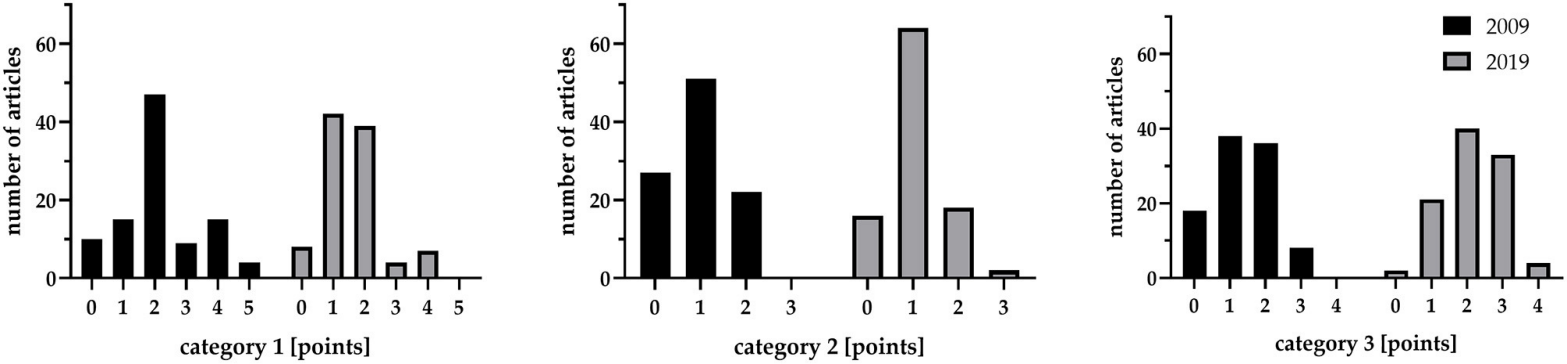
Quality score according to the three analyzed categories: “anesthesia monitoring”–category 1, “ischemia verification”–category 2 and “general quality standards”–category 3.

The mean quality score was 4.45, respectively, 4.82 (for 2009/2019; *p* = 0.243). In category 1 “anesthesia monitoring” the studies from 2009 outperformed 2019 (2.16 vs. 1.60, *p* < 0.001), whereas in category 2 (*p* = 0.300) and 3 (*p* < 0.001) the studies from 2019 showed higher results (2019: 1.06 and 2.16 vs. 2009: 0.95 and 1.34). The data is presented in Figure 2.

Both for 2009 and 2019, quality scores did not differ dependent on the continent of origin (*p* = 0.087; *p* = 0.171).

With respect to study type (any study examining an intervention with regards to its impact on the ischemia was defined as treatment study), 32 studies used an explorative approach, whereas 168 studies evaluated a treatment. The assessed quality score did not differ significantly between exploratory and treatment studies (mean ± SD: 4.28 ± 1.63 vs. 4.70 ± 1.88; *p* = 0.282). Considering only the studies from 2009, again, no significant difference was seen (exploratory vs. treatment: 4.44 ± 2.06 vs. 4.45 ± 2.01; *p* = 0.954), whereas for the studies from 2019 a trend toward a higher quality score in the treatment group (4.13 ± 1.09 vs. 4.95 ± 1.71; *p* = 0.057).

## Discussion

Our results indicate that an inadequate experimental performance itself namely a lack of sufficient quality control (in terms of ischemia verification and exclusion of undesired pathologies) still is common. The reporting of some methodological aspects (such as the reporting of approval) has increased over time. However, studies largely lack an a priori sample size calculation and other essential items indicating methodological quality. Unfortunately, same applies for other experimental issues (besides ischemia verification) such as periinterventional monitoring of physiological parameters. Thus, despite the publication of the STAIR guideline in 2009 and the original ARRIVE guidelines in 2010 there is little improvement with respect to methodology/reporting.

Stroke is a common neurological disorder resulting in a major socioeconomic burden. Thus, neuroscientific efforts largely have been focused on neuroprotective therapies after stroke. However, preclinical stroke research has proven to be impressively unsuccessful when it comes to clinical translation (7). As said earlier, various guidelines have been developed in order to improve the design quality of these studies, the STAIR criteria being one of the most famous and acknowledged ones (9). However, the STAIR criteria cover only some aspects of the methodology and experimental procedure; some recommendations are expressed only vaguely. CBF measurement (or perfusion imaging) is considered to be an important measure for adequate occlusion (9). Various studies have pointed out the relevance of (bilateral) CBF measurement as a gold standard for occlusion verification but also for detection of undesired events (such as SAH or premature reperfusion) (17, 18). Unfortunately, the majority of studies analyzed still lacks a documentation of CBF measurement (both for the samples from 2009 and 2019). It is disappointing that even in 2019, when the guideline had been widely approved, only roughly one third of the studies analyzed reported a method of CBF measurement. Compared to our sample from 2009, when suggestions from the STAIR guideline had not yet been available, the overall usage of CBF measurement has not improved.

In 2009, Philip and colleagues have already demonstrated the poor methodological quality of preclinical stroke studies pointing out the importance of CBF measurement for the reliability of the experimental result (23). However, our results indicate that there is little improvement over time with respect to CBF monitoring. Notably, bilateral CBF measurement explicitly was mentioned in only two cases (out of 100 articles published in 2019). It is not clear why application of CBF measurement is that scarce as its advantage has already been demonstrated. Availability of other methods (such as MR imaging) may be regarded as substitutes. To some extent, this applies for MR imaging, as ischemia (and its extent) is verified and undesired pathologies are ruled out in parallel. Though, MR imaging rarely is used (10 and 12% for 2009/2019) and other methods for ischemia verification mainly suffer from its retrospective character. Thus, entities like SAH may be overseen when methods such as postmortem inspection or TTC staining are applied ex post. Same applies for neuroscores, which are not a reliable instrument, as they do not reflect the extent of ischemia nor rule out complications such as SAH. Further, although usually hemiparesis is observed, it is not possible to attribute the neurological deficit to a small strategic ischemia (within the basal ganglia) or a large hemispheric infarction.

Lesion patterns also may vary due to different filaments used (26). Our data show that the material used varies largely, which may be a contributing factor to heterogeneity of experimental results. Additionally, definitions for “Sham” vary substantially between the studies (surgery without filament insertion, surgery with filament insertion, no surgery at all).

Further, periinterventional monitoring of physiological parameters in general was reported less common in the articles analyzed from 2019 compared with those from 2009 although the influence of anesthesia and its monitoring for the experimental result has been demonstrated repeatedly (22, 27). Similarly, Thomas and colleagues detected no improvement in reporting of periinterventional parameters (such as ventilation, blood gas analysis, end-tidal carbon dioxide concentration, blood pressure, administration of intravenous fluids or analgesics) comparing experimental studies from 2005 and 2015 regarding the adherence of the STAIR guideline (28). This is an astonishing fact, as the STAIR guideline (9) but also others (11) recommend the monitoring explicitly. It is unclear (and only speculative) if this development is due to an increase in “publication pressure” resulting in easier and less elaborated (less expensive) experimental setups (29). The journal-specific word limit may also represent a further-seemingly trivial-obstacle avoiding detailed reporting. However, most journals offer the publication of supplement data to allow complete reporting.

Another issue of preclinical research (and research in general) is the methodological aspect. General guidelines like ARRIVE (14, 15) promote the implementation of certain methodological quality standards. Further, specific guidelines like IMPROVE (13) as well as elaborated manuals and critical reviews of rodent stroked modeling (11, 23, 30, 31) emphasize the importance of a sophisticated planning, adequate performance, and transparent reporting of experimental studies in order to create a relevant experimental result. Some general aspects of methodology (like mention of approval by authorities and randomization) have improved over time analyzing 100 samples from 2009 vs. 2019. However, essential facets of methodology still are rarely reported (with only little improvement between 2009 and 2019): Particularly the documentation of an a priori sample size calculation is scarce; further, the studies often lack a clear declaration of in-/exclusion criteria. Given that an a priori sample size calculation presents the essential fundament of most study designs, the current results are not comprehensible. Most guidelines explicitly point out the importance of sample size calculation. Thus, the negligence of this issue most probably is not a matter of ignorance. The lack of underlying data allowing a proper sample size calculation may take part, but, again, “publication pressure” also may play a role as experimental performance is facilitated by low sample sizes. “Historical” sample sizes (such as treatment groups of three or five animals) usually allow a much faster processing of the experiments, whereas realistic effect sizes regularly result in high sample sizes and, thus, a lengthy workflow. Low sample size very often leads to statistically underpowered studies, not being able to detect true effects or, when finding a significant result, producing inflated estimates of the true effect. Besides the scientific implication, underpowered studies and thus unreliable results also imply an ethical dimension concerning the wasting use of animals in scientific research (32). In general, risk of bias seems to be a major issue in experimental studies, which is not only limited to preclinical stroke research (33).

However, a guideline (such as ARRIVE and STAIR) is not necessarily a one-fits-all concept. ARRIVE, for example, is focused on RCTs and some items may not apply for exploratory studies. Notwithstanding, elementary methodological standards should be generally implemented. Thus, blind adherence to a specific guideline is certainly not desirable, but a deliberated usage including an honest discussion on reasons why certain criteria have not been complied with is needed.

Another pitfall of preclinical stroke research is the experimental focus on young, male rats instead of sex-mixed groups, aged, or hypertensive animals (31, 34–37). With respect to sex it has been proven, that inclusion of both sexes is highly desirable as it decreases bias of the results and is not necessarily accompanied by increase of sample size (38–40).

However, the vast majority of animals still are young, male rats (both in our samples from 2009 and 2019). Thus, both the underlying comorbidity/fundament of the pathology is not represented and the possible influence of sex on the specific course is not taken into account. Further, current research emphasizes the importance of the circadian rhythm even for preclinical stroke research (41). Therefore, the reporting of the exact time of day may contribute an important aspect of reporting in the future.

Consistent with the results from Thomas and colleagues (28), a substantial increase of publications from Asia (particularly from China) in parallel to a decrease of studies from North America was noted. The finding is in line with other data not only focusing on stroke research (42), but the predominance of studies from China (67% in 2019) compared to other data is striking (28, 42, 43). However, these are descriptive results. We have not correlated the country (or continent) of origin with methodological quality. Further, it has not been assessed, whether “established” stroke labs comply more or less with the current guidelines. Both scenarios are conceivable, either due to available know-how by an experienced team or to plugged-in patterns (no longer able to change).

In conclusion, our presented data show that the procedure itself varies largely resulting in heterogeneous lesion patterns and that both specific important aspects of experimental procedure (such as ischemia verification) and methodological issues are still in need of improvement. There is some change over time particularly with respect to reporting of approval and randomization but both study planning and the procedure itself still are prone to biases. This is not only a technical and formal issue because the results itself are influenced by the factors already mentioned. It is rather an essential and fundamental point as the relevance of the experimental result depends on adequate methodology and modeling. Further, it is a matter of appropriate resource management, animal rights and, finally, scientific ethics. Various articles have already pointed out the general importance of methodology and transparency in reporting (44–46). Our data show, that with respect to preclinical stroke research, there is still a long way to go. In addition, procedure-specific quality aspects (such as the essential aspect of ischemia verification and exclusion of other pathologies in parallel) are widely not applied and/or not reported. Thus, there are several methodological and procedural factors, which may contribute to the translational failure of stroke research. It is therefore important to identify the reasons for the pattern and to develop strategies, which may improve the quality of preclinical stroke research and standardize the disease model. Several journals nowadays demand a confirmation of the authors that current guidelines have been implemented. However, the adherence to the guidelines itself rarely is verified in detail. As a solution “*…mandatory reporting of key methodological parameters in the published article and not only during submission”* has already been proposed by a group analyzing articles on experimental stroke published in *Stroke* (16). Further, not only general aspects have to be taken into account but also procedure-specific ones. With respect to stroke research, it is commonly referred to the STAIR criteria, which build an excellent framework but lack some detailed advice (11). Finally, it is a matter of science funding and policy to set the agenda for elaborated study designs and procedures. As things have hardly changed so far, it is indispensable to put some pressure on the system. This does not imply a “science police” chasing non-adherent authors, but first of all it implies the willingness of the entire scientific system to change and, further, it implies adequate measures. Quality scores (as exemplarily presented) may be part of the solution in order to facilitate the process for reviewers and editors, but may be too rigid for specific settings. Another important issue is to educate younger scientist accordingly and to encourage a critical scrutiny. Lastly, funders and institutions should adopt a culture which does not only value the mere quantity of publications but also the methodological quality.

That said, it is definitively a long way to go and a real transformation process will be dependent on the willingness and engagement of the entire scientific community. The reasons for disregarding well-approved guidelines are not obvious and most probably may be multifactorial. On the one hand, there seems to be a lack of pressure from institutions/ funders/journals to implement certain quality standards and guideline adherence. Further, historical conditions, human phlegm, sometimes nescience or absence of (financial and timely) resources may contribute to the lack of reporting and experimental quality. However, it has to be assumed that anyone in preclinical science is honestly eager for improvement of the own and the general scientific quality. Therefore, any effort toward high-grade methodology and transparency has to be appreciated from the community and the entire scientific system. Emphasizing the importance of a highest possible scientific quality as well as repetitive education are important tools to raise the public awareness. Besides, it is necessary to repetitively analyze the current status quo of preclinical research in order to detect deficiencies and develop strategies to overcome the broad resistance.

Of note, our study has some limitations: Only one database for literature search was used. However, our goal was not to fully evaluate the literature on experimental stroke research in the rat model for the years studied but to give an adequate overview of the trends in general. Thus, we decided to analyze 100 publications for each year (2009 and 2019) sorted in ascending order according to their publication date as a representative subset. As only 100 publications per year have been analyzed, a random accumulation of methodologically inadequate studies in 1 year compared with the other may have biased the results. However, if the publications of an entire year would have been evaluated arising bias cannot be excluded but may be less probable. In order to evaluate the longitudinal awareness to methodological quality aspects and the adherence to current guidelines, a prospective study would be reasonable.

Further, only two time-points were evaluated. This approach was chosen in order to depict the status quo of preclinical stroke research using the MCAO model before (2009) and after (2019) the public awareness of STAIR and ARRIVE. A further (third) time-point would have allowed to depict a trend over time. Data extraction and analyses were performed by only one person, but it was validated by a second author and there was limited space for subjective interpretation due to the clearly defined parameters in advance. We defined neuroscore assessment after treatment as a doubtful indicator for ischemia evaluation (due to the supposed effect of treatment on neuroscore). CBF measurement might also be influenced by a prior treatment; however, if used as ischemia verification usually clear cut-offs were given, thus, representing an objective indicator for a similar cerebral underperfusion. Further, like any other comparable score with ordinal data, our quality score is subjected to general limitations. In most categories, we have chosen a binary scoring system, whereby the corresponding parameters are all equally rated in terms of their influence on study quality. Only in the case of CBF measurement we chose a ternary decision.

## Conclusions

The reporting of periinterventional parameters in experimental stroke research (particularly, the one in search for neuroprotective agents) using the MCAO model still is scarce. Some methodological aspects have improved over time (2009 compared with 2019), but essential issues (such as sample size calculations) are reported rarely. Thus, deficits in the methodological and procedural quality may contribute to the translational failure of preclinical stroke research.

## Data Availability Statement

The raw data is available from the corresponding author on request.

## Ethics Statement

Ethical review and approval was not required for the animal study because of secondary analyses of animal experiments.

## Author Contributions

AH: design of the study. JF and AH: data extraction, analysis, and visualization. JF: first draft of the manuscript. AH and UL: revising of the manuscript. JF, UL, and AH: final approval and interpretation of the data. All authors contributed to the article and approved the submitted version.

## Funding

UL: German Research Foundation (Deutsche Forschungsgemeinschaft–DFG, grant number LI 588/5-1, LI 588/5-2) as part of the German research unit Severity assessment in animal based research FOR 2591. AH: B. Braun-Stiftung EMID: 43da889f91b25360.

## Conflict of Interest

The authors declare that the research was conducted in the absence of any commercial or financial relationships that could be construed as a potential conflict of interest.

## Publisher's Note

All claims expressed in this article are solely those of the authors and do not necessarily represent those of their affiliated organizations, or those of the publisher, the editors and the reviewers. Any product that may be evaluated in this article, or claim that may be made by its manufacturer, is not guaranteed or endorsed by the publisher.
