# Supplementary material for: Procedural and Methodological Quality in Preclinical Stroke Research–A Cohort Analysis of the Rat MCAO Model Comparing Periods Before and After the Publication of STAIR/ARRIVE
Source: Front Neurol. 2022 May 30;13:834003. doi: 10.3389/fneur.2022.834003 (PMC9190283; doi:10.3389/fneur.2022.834003)
Supplement: Supplementary file 1 [file Table_1.DOCX]

Supplementary Material

**Supplementary reference list for 2009**

This supplementary reference list includes all publications that were included in the analysis for the year 2009.

1. Zhong S, Li Z, Huan L, Chen BY. Neurochemical Mechanism of Electroacupuncture: Anti-injury Effect on Cerebral Function after Focal Cerebral Ischemia in Rats. Evid Based Complement Alternat Med. 2009 Mar;6(1):51-6. doi: 10.1093/ecam/nem062. Epub 2007 Oct 27. PMID: 18955263; PMCID: PMC2644276.
2. Chen J, Cui X, Zacharek A, Chopp M. Increasing Ang1/Tie2 expression by simvastatin treatment induces vascular stabilization and neuroblast migration after stroke. J Cell Mol Med. 2009 Jul;13(7):1348-57. doi: 10.1111/j.1582-4934.2008.00380.x. Epub 2008 Jun 9. PMID: 18544044; PMCID: PMC3710660.
3. Wang SH, Zhang ZJ, Guo YJ, Zhou H, Teng GJ, Chen BA. Anhedonia and activity deficits in rats: impact of post-stroke depression. J Psychopharmacol. 2009 May;23(3):295-304. doi: 10.1177/0269881108089814. Epub 2008 Jun 18. PMID: 18562439.
4. Modo M, Beech JS, Meade TJ, Williams SC, Price J. A chronic 1 year assessment of MRI contrast agent-labelled neural stem cell transplants in stroke. Neuroimage. 2009 Aug;47 Suppl 2(0 2):T133-42. doi: 10.1016/j.neuroimage.2008.06.017. Epub 2008 Jun 25. PMID: 18634886; PMCID: PMC4145694.
5. Li Q, Zhang R, Guo YL, Mei YW. Effect of neuregulin on apoptosis and expressions of STAT3 and GFAP in rats following cerebral ischemic reperfusion. J Mol Neurosci. 2009 Jan;37(1):67-73. doi: 10.1007/s12031-008-9121-3. Epub 2008 Jul 17. PMID: 18633737.
6. Zhao WH, Ji XM, Ling F, Ding YC, Xing CH, Wu H, Guo M, Xuan Y, Guan B, Jiang LL. Local mild hypothermia induced by intra-arterial cold saline infusion prolongs the time window of onset of reperfusion injury after transient focal ischemia in rats. Neurol Res. 2009 Feb;31(1):43-51. doi: 10.1179/174313208X327982. Epub 2008 Aug 7. PMID: 18691447.
7. Zhu XL, Xiong LZ, Wang Q, Liu ZG, Ma X, Zhu ZH, Hu S, Gong G, Chen SY. Therapeutic time window and mechanism of tetramethylpyrazine on transient focal cerebral ischemia/reperfusion injury in rats. Neurosci Lett. 2009 Jan 2;449(1):24-7. doi: 10.1016/j.neulet.2008.09.007. Epub 2008 Sep 6. PMID: 18790005.
8. Badin RA, Modo M, Cheetham M, Thomas DL, Gadian DG, Latchman DS, Lythgoe MF. Protective effect of post-ischaemic viral delivery of heat shock proteins in vivo. J Cereb Blood Flow Metab. 2009 Feb;29(2):254-63. doi: 10.1038/jcbfm.2008.106. Epub 2008 Sep 10. PMID: 18781161; PMCID: PMC2702130.
9. Li Q, Huang XJ, He W, Ding J, Jia JT, Fu G, Wang HX, Guo LJ. Neuroprotective potential of fasudil mesylate in brain ischemia-reperfusion injury of rats. Cell Mol Neurobiol. 2009 Mar;29(2):169-80. doi: 10.1007/s10571-008-9308-8. Epub 2008 Sep 11. PMID: 18785000.
10. Jokivarsi KT, Niskanen JP, Michaeli S, Gröhn HI, Garwood M, Kauppinen RA, Gröhn OH. Quantitative assessment of water pools by T 1 rho and T 2 rho MRI in acute cerebral ischemia of the rat. J Cereb Blood Flow Metab. 2009 Jan;29(1):206-16. doi: 10.1038/jcbfm.2008.113. Epub 2008 Oct 1. PMID: 18827834; PMCID: PMC4783795.
11. Li Q, Zhang R, Ge YL, Mei YW, Guo YL. Effects of neuregulin on expression of MMP-9 and NSE in brain of ischemia/reperfusion rat. J Mol Neurosci. 2009 Jun;38(2):207-15. doi: 10.1007/s12031-008-9150-y. Epub 2008 Oct 2. PMID: 18830828.
12. Foster KA, Regan HK, Danziger AP, Detwiler T, Kwon N, Rickert K, Lynch JJ, Regan CP. Attenuation of edema and infarct volume following focal cerebral ischemia by early but not delayed administration of a novel small molecule KDR kinase inhibitor. Neurosci Res. 2009 Jan;63(1):10-6. doi: 10.1016/j.neures.2008.09.007. Epub 2008 Oct 4. PMID: 18951929.
13. Osmond JM, Dorrance AM. 11beta-hydroxysteroid dehydrogenase type II inhibition causes cerebrovascular remodeling and increases infarct size after cerebral ischemia. Endocrinology. 2009 Feb;150(2):713-9. doi: 10.1210/en.2008-0808. Epub 2008 Oct 9. PMID: 18845645; PMCID: PMC2646544.
14. Zacharek A, Chen J, Cui X, Yang Y, Chopp M. Simvastatin increases notch signaling activity and promotes arteriogenesis after stroke. Stroke. 2009 Jan;40(1):254-60. doi: 10.1161/STROKEAHA.108.524116. Epub 2008 Oct 16. PMID: 18927449; PMCID: PMC2804086.
15. Xu L, Voloboueva LA, Ouyang Y, Emery JF, Giffard RG. Overexpression of mitochondrial Hsp70/Hsp75 in rat brain protects mitochondria, reduces oxidative stress, and protects from focal ischemia. J Cereb Blood Flow Metab. 2009 Feb;29(2):365-74. doi: 10.1038/jcbfm.2008.125. Epub 2008 Nov 5. PMID: 18985056; PMCID: PMC3676940.
16. Henninger N, Bouley J, Bråtane BT, Bastan B, Shea M, Fisher M. Laser Doppler flowmetry predicts occlusion but not tPA-mediated reperfusion success after rat embolic stroke. Exp Neurol. 2009 Feb;215(2):290-7. doi: 10.1016/j.expneurol.2008.10.013. Epub 2008 Nov 7. PMID: 19038254.
17. Yousuf S, Atif F, Ahmad M, Hoda N, Ishrat T, Khan B, Islam F. Resveratrol exerts its neuroprotective effect by modulating mitochondrial dysfunctions and associated cell death during cerebral ischemia. Brain Res. 2009 Jan 23;1250:242-53. doi: 10.1016/j.brainres.2008.10.068. Epub 2008 Nov 11. PMID: 19027723.
18. Cheng J, Hu W, Toung TJ, Zhang Z, Parker SM, Roselli CE, Hurn PD. Age- dependent effects of testosterone in experimental stroke. J Cereb Blood Flow Metab. 2009 Mar;29(3):486-94. doi: 10.1038/jcbfm.2008.138. Epub 2008 Nov 12. PMID: 19002196; PMCID: PMC2843561.
19. Kim WS, Kim IS, Kim SJ, Wei P, Hyung Choi D, Han TR. Effect of electroacupuncture on motor recovery in a rat stroke model during the early recovery stage. Brain Res. 2009 Jan 12;1248:176-83. doi: 10.1016/j.brainres.2008.11.009. Epub 2008 Nov 13. PMID: 19041635.
20. Li H, Deng CQ, Chen BY, Zhang SP, Liang Y, Luo XG. Total saponins of Panax notoginseng modulate the expression of caspases and attenuate apoptosis in rats following focal cerebral ischemia-reperfusion. J Ethnopharmacol. 2009 Jan 30;121(3):412-8. doi: 10.1016/j.jep.2008.10.042. Epub 2008 Nov 18. PMID: 19059471.
21. Simard JM, Yurovsky V, Tsymbalyuk N, Melnichenko L, Ivanova S, Gerzanich V. Protective effect of delayed treatment with low-dose glibenclamide in three models of ischemic stroke. Stroke. 2009 Feb;40(2):604-9. doi: 10.1161/STROKEAHA.108.522409. Epub 2008 Nov 20. PMID: 19023097; PMCID: PMC2744391.
22. Zhang HF, Hu XM, Wang LX, Xu SQ, Zeng FD. Protective effects of scutellarin against cerebral ischemia in rats: evidence for inhibition of the apoptosis- inducing factor pathway. Planta Med. 2009 Feb;75(2):121-6. doi: 10.1055/s-0028-1088368. Epub 2008 Nov 24. PMID: 19031363.
23. Hu Q, Chen C, Yan J, Yang X, Shi X, Zhao J, Lei J, Yang L, Wang K, Chen L, Huang H, Han J, Zhang JH, Zhou C. Therapeutic application of gene silencing MMP-9 in a middle cerebral artery occlusion-induced focal ischemia rat model. Exp Neurol. 2009 Mar;216(1):35-46. doi: 10.1016/j.expneurol.2008.11.007. Epub 2008 Nov 24. PMID: 19073180.
24. Toyama K, Honmou O, Harada K, Suzuki J, Houkin K, Hamada H, Kocsis JD. Therapeutic benefits of angiogenetic gene-modified human mesenchymal stem cells after cerebral ischemia. Exp Neurol. 2009 Mar;216(1):47-55. doi: 10.1016/j.expneurol.2008.11.010. Epub 2008 Nov 27. PMID: 19094989.
25. Matsuda S, Umeda M, Kato H, Araki T. Glial damage after transient focal cerebral ischemia in rats. J Mol Neurosci. 2009 Jun;38(2):220-6. doi: 10.1007/s12031-008-9165-4. Epub 2008 Dec 3. PMID: 19051061.
26. Wang T, Fu FH, Han B, Zhu M, Yu X, Zhang LM. Aspirin attenuates cerebral ischemic injury in diabetic rats. Exp Clin Endocrinol Diabetes. 2009 Apr;117(4):181-5. doi: 10.1055/s-0028-1087210. Epub 2008 Dec 3. PMID: 19053022.
27. Abrahams JM, Lenart CJ, Tobias ME. Temporal variation of induction neurogenesis in a rat model of transient middle cerebral artery occlusion. Neurol Res. 2009 Jun;31(5):528-33. doi: 10.1179/174313209X380801. Epub 2008 Dec 3. PMID: 19055874.
28. Belayev L, Khoutorova L, Atkins K, Cherqui A, Alvarez-Builla J, Bazan NG. LAU-0901, a novel platelet-activating factor receptor antagonist, confers enduring neuroprotection in experimental focal cerebral ischemia in the rat. Brain Res. 2009 Feb 9;1253:184-90. doi: 10.1016/j.brainres.2008.11.074. Epub 2008 Dec 3. PMID: 19070607; PMCID: PMC2637461.
29. Regan C, Shepherd C, Strack A, Weinberg D, Nargund R, Ye Z, Pollard P, Fong T, Reynolds I, Lynch J. Lack of protection with a novel, selective melanocortin receptor subtype-4 agonist RY767 in a rat transient middle cerebral artery occlusion stroke model. Pharmacology. 2009;83(1):38-44. doi: 10.1159/000178811. Epub 2008 Dec 4. PMID: 19052481.
30. Cheng S, Ma M, Ma Y, Wang Z, Xu G, Liu X. Combination therapy with intranasal NGF and electroacupuncture enhanced cell proliferation and survival in rats after stroke. Neurol Res. 2009 Sep;31(7):753-8. doi: 10.1179/174313209X382557. Epub 2008 Dec 5. PMID: 19061539.
31. Jensen UR, Liu JR, Eschenfelder C, Meyne J, Zhao Y, Deuschl G, Jansen O, Ulmer S. The correlation between quantitative T2' and regional cerebral blood flow after acute brain ischemia in early reperfusion as demonstrated in a middle cerebral artery occlusion/reperfusion model of the rat. J Neurosci Methods. 2009 Mar 30;178(1):55-8. doi: 10.1016/j.jneumeth.2008.11.023. Epub 2008 Dec 6. PMID: 19103223.
32. He Y, Hua Y, Liu W, Hu H, Keep RF, Xi G. Effects of cerebral ischemia on neuronal hemoglobin. J Cereb Blood Flow Metab. 2009 Mar;29(3):596-605. doi: 10.1038/jcbfm.2008.145. Epub 2008 Dec 10. PMID: 19066615; PMCID: PMC2683405.
33. Yagi K, Kitazato KT, Uno M, Tada Y, Kinouchi T, Shimada K, Nagahiro S. Edaravone, a free radical scavenger, inhibits MMP-9-related brain hemorrhage in rats treated with tissue plasminogen activator. Stroke. 2009 Feb;40(2):626-31. doi: 10.1161/STROKEAHA.108.520262. Epub 2008 Dec 18. PMID: 19095969.
34. Renic M, Klaus JA, Omura T, Kawashima N, Onishi M, Miyata N, Koehler RC, Harder DR, Roman RJ. Effect of 20-HETE inhibition on infarct volume and cerebral blood flow after transient middle cerebral artery occlusion. J Cereb Blood Flow Metab. 2009 Mar;29(3):629-39. doi: 10.1038/jcbfm.2008.156. Epub 2008 Dec 24. PMID: 19107134; PMCID: PMC2821901.
35. Paik MJ, Li WY, Ahn YH, Lee PH, Choi S, Kim KR, Kim YM, Bang OY, Lee G. The free fatty acid metabolome in cerebral ischemia following human mesenchymal stem cell transplantation in rats. Clin Chim Acta. 2009 Apr;402(1-2):25-30. doi: 10.1016/j.cca.2008.12.022. Epub 2008 Dec 25. PMID: 19161994.
36. Chen C, Hu Q, Yan J, Yang X, Shi X, Lei J, Chen L, Huang H, Han J, Zhang JH, Zhou C. Early inhibition of HIF-1alpha with small interfering RNA reduces ischemic-reperfused brain injury in rats. Neurobiol Dis. 2009 Mar;33(3):509-17. doi: 10.1016/j.nbd.2008.12.010. Epub 2008 Dec 31. PMID: 19166937.
37. Hua Q, Zhu XL, Li PT, Liu Y, Zhang N, Xu Y, Jia X. The inhibitory effects of cholalic acid and hyodeoxycholalic acid on the expression of TNFalpha and IL-1beta after cerebral ischemia in rats. Arch Pharm Res. 2009 Jan;32(1):65-73. doi: 10.1007/s12272-009-1119-z. Epub 2009 Jan 29. PMID: 19183878.
38. Popp A, Jaenisch N, Witte OW, Frahm C. Identification of ischemic regions in a rat model of stroke. PLoS One. 2009;4(3):e4764. doi: 10.1371/journal.pone.0004764. Epub 2009 Mar 10. PMID: 19274095; PMCID: PMC2652027.
39. Zawadzka M, Lukasiuk K, Machaj EK, Pojda Z, Kamińska B. Lack of migration and neurological benefits after infusion of umbilical cord blood cells in ischemic brain injury. Acta Neurobiol Exp (Wars). 2009;69(1):46-51. PMID: 19325640.
40. Wang L, Sun L, Zhang Y, Wu H, Li C, Pan Z, Lu Y, Yang B. Ionic mechanisms underlying action potential prolongation by focal cerebral ischemia in rat ventricular myocytes. Cell Physiol Biochem. 2009;23(4-6):305-16. doi: 10.1159/000218177. Epub 2009 May 6. PMID: 19471098.
41. Daadi MM, Lee SH, Arac A, Grueter BA, Bhatnagar R, Maag AL, Schaar B, Malenka RC, Palmer TD, Steinberg GK. Functional engraftment of the medial ganglionic eminence cells in experimental stroke model. Cell Transplant. 2009;18(7):815-26. doi: 10.3727/096368909X470829. Epub 2009 Apr 15. PMID: 19500468.
42. Sohn Y, Kang HC, Kim KS, Park SM, Sohn NW, Jung HS, Kim SH. Protective effects of natrii sulfas on cerebral focal ischemia induced by MCAO in rats. Am J Chin Med. 2009;37(2):273-93. doi: 10.1142/S0192415X09006849. PMID: 19507272.
43. Yang KL, Chen MF, Liao CH, Pang CY, Lin PY. A simple and efficient method for generating Nurr1-positive neuronal stem cells from human wisdom teeth (tNSC) and the potential of tNSC for stroke therapy. Cytotherapy. 2009;11(5):606-17. doi: 10.1080/14653240902806994. PMID: 19579137.
44. Cho JH, Sung JH, Cho EH, Won CK, Lee HJ, Kim MO, Koh PO. Gingko biloba Extract (EGb 761) prevents ischemic brain injury by activation of the Akt signaling pathway. Am J Chin Med. 2009;37(3):547-55. doi: 10.1142/S0192415X09007041. PMID: 19606514.
45. Amantea D, Marrone MC, Nisticò R, Federici M, Bagetta G, Bernardi G, Mercuri NB. Oxidative stress in stroke pathophysiology validation of hydrogen peroxide metabolism as a pharmacological target to afford neuroprotection. Int Rev Neurobiol. 2009;85:363-74. doi: 10.1016/S0074-7742(09)85025-3. PMID: 19607981.
46. Amantea D, Fratto V, Maida S, Rotiroti D, Ragusa S, Nappi G, Bagetta G, Corasaniti MT. Prevention of Glutamate Accumulation and Upregulation of Phospho- Akt may Account for Neuroprotection Afforded by Bergamot Essential Oil against Brain Injury Induced by Focal Cerebral Ischemia in Rat. Int Rev Neurobiol. 2009;85:389-405. doi: 10.1016/S0074-7742(09)85027-7. PMID: 19607983.
47. Gonzalez FF, Abel R, Almli CR, Mu D, Wendland M, Ferriero DM. Erythropoietin sustains cognitive function and brain volume after neonatal stroke. Dev Neurosci. 2009;31(5):403-11. doi: 10.1159/000232558. Epub 2009 Aug 11. PMID: 19672069; PMCID: PMC2820334.
48. Nakano T, Okamoto H. Dexmedetomidine-induced cerebral hypoperfusion exacerbates ischemic brain injury in rats. J Anesth. 2009;23(3):378-84. doi: 10.1007/s00540-009-0777-9. Epub 2009 Aug 14. PMID: 19685118.
49. Detante O, Moisan A, Dimastromatteo J, Richard MJ, Riou L, Grillon E, Barbier E, Desruet MD, De Fraipont F, Segebarth C, Jaillard A, Hommel M, Ghezzi C, Remy C. Intravenous administration of 99mTc-HMPAO-labeled human mesenchymal stem cells after stroke: in vivo imaging and biodistribution. Cell Transplant. 2009;18(12):1369-79. doi: 10.3727/096368909X474230. Epub 2009 Sep 28. PMID: 19849895.
50. Koh PO. Gingko biloba extract (EGb 761) prevents increase of Bad-Bcl-XL interaction following cerebral ischemia. Am J Chin Med. 2009;37(5):867-76. doi: 10.1142/S0192415X0900734X. PMID: 19885947.
51. Yamamoto Y, Yanagisawa M, Tak NW, Watanabe K, Takahashi C, Fujisawa A, Kashiba M, Tanaka M. Repeated edaravone treatment reduces oxidative cell damage in rat brain induced by middle cerebral artery occlusion. Redox Rep. 2009;14(6):251-8. doi: 10.1179/135100009X12525712409779. PMID: 20003710.
52. Yanagida T, Tsushima J, Kitamura Y, Yanagisawa D, Takata K, Shibaike T, Yamamoto A, Taniguchi T, Yasui H, Taira T, Morikawa S, Inubushi T, Tooyama I, Ariga H. Oxidative stress induction of DJ-1 protein in reactive astrocytes scavenges free radicals and reduces cell injury. Oxid Med Cell Longev. 2009 Jan- Mar;2(1):36-42. doi: 10.4161/oxim.2.1.7985. PMID: 20046643; PMCID: PMC2763229.
53. Fan H, Li L, Zhang X, Liu Y, Yang C, Yang Y, Yin J. Oxymatrine downregulates TLR4, TLR2, MyD88, and NF-kappaB and protects rat brains against focal ischemia. Mediators Inflamm. 2009;2009:704706. doi: 10.1155/2009/704706. Epub 2010 Feb 16. PMID: 20182634; PMCID: PMC2825667.
54. Kavakli A, Kose E, Akpolat N, Sarsilmaz M. The effects of acupuncture on rats with brain ischemia-reperfusion. Neurosciences (Riyadh). 2009 Jan;14(1):10-3. PMID: 21048566.
55. Yu Y, Luo X, Ren QG, Yi CJ, Yu ZY, Xie XW, Wang W. The involvement of upregulation and translocation of phospho-Rb in early neuronal apoptosis following focal cerebral ischemia in rats. Neurochem Res. 2009 Jun;34(6):1113-9. doi: 10.1007/s11064-008-9887-2. Epub 2009 Jan 4. PMID: 19123049.
56. Jia J, Zhang X, Hu YS, Wu Y, Wang QZ, Li NN, Wu CQ, Yu HX, Guo QC. Protective effect of tetraethyl pyrazine against focal cerebral ischemia/reperfusion injury in rats: therapeutic time window and its mechanism. Thromb Res. 2009 Mar;123(5):727-30. doi: 10.1016/j.thromres.2008.11.004. Epub 2009 Jan 6. PMID: 19128823.
57. Zhang P, Li J, Liu Y, Chen X, Kang Q. Transplanted human embryonic neural stem cells survive, migrate, differentiate and increase endogenous nestin expression in adult rat cortical peri-infarction zone. Neuropathology. 2009 Aug;29(4):410-21. doi: 10.1111/j.1440-1789.2008.00993.x. Epub 2009 Jan 6. PMID: 19170896.
58. Lu A, Clark JF, Broderick JP, Pyne-Geithman GJ, Wagner KR, Khatri P, Tomsick T, Sharp FR. Mechanical reperfusion is associated with post-ischemic hemorrhage in rat brain. Exp Neurol. 2009 Apr;216(2):407-12. doi: 10.1016/j.expneurol.2008.12.020. Epub 2009 Jan 7. PMID: 19162014; PMCID: PMC2659349.
59. Bigdeli MR. Preconditioning with prolonged normobaric hyperoxia induces ischemic tolerance partly by upregulation of antioxidant enzymes in rat brain tissue. Brain Res. 2009 Mar 13;1260:47-54. doi: 10.1016/j.brainres.2008.12.065. Epub 2009 Jan 7. Erratum in: Brain Res. 2009 Jul 24;1281:128-9. PMID: 19167368.
60. Anan M, Abe T, Shimotaka K, Kamida T, Kubo T, Fujiki M, Kobayashi H. Induction of collateral circulation by hypoxia-inducible factor 1alpha decreased cerebral infarction in the rat. Neurol Res. 2009 Nov;31(9):917-22. doi: 10.1179/174313209X383231. Epub 2009 Jan 9. PMID: 19138472.
61. Kuge A, Takemura S, Kokubo Y, Sato S, Goto K, Kayama T. Temporal profile of neurogenesis in the subventricular zone, dentate gyrus and cerebral cortex following transient focal cerebral ischemia. Neurol Res. 2009 Nov;31(9):969-76. doi: 10.1179/174313209X383312. Epub 2009 Jan 9. PMID: 19138475.
62. Matchett GA, Fathali N, Hasegawa Y, Jadhav V, Ostrowski RP, Martin RD, Dorotta IR, Sun X, Zhang JH. Hydrogen gas is ineffective in moderate and severe neonatal hypoxia-ischemia rat models. Brain Res. 2009 Mar 9;1259:90-7. doi: 10.1016/j.brainres.2008.12.066. Epub 2009 Jan 10. PMID: 19168038.
63. Li JS, Zhang W, Kang ZM, Ding SJ, Liu WW, Zhang JH, Guan YT, Sun XJ. Hyperbaric oxygen preconditioning reduces ischemia-reperfusion injury by inhibition of apoptosis via mitochondrial pathway in rat brain. Neuroscience. 2009 Apr 10;159(4):1309-15. doi: 10.1016/j.neuroscience.2009.01.011. Epub 2009 Jan 13. PMID: 19185051.
64. Dharap A, Bowen K, Place R, Li LC, Vemuganti R. Transient focal ischemia induces extensive temporal changes in rat cerebral microRNAome. J Cereb Blood Flow Metab. 2009 Apr;29(4):675-87. doi: 10.1038/jcbfm.2008.157. Epub 2009 Jan 14. PMID: 19142192; PMCID: PMC2743462.
65. Yamashita T, Kamiya T, Deguchi K, Inaba T, Zhang H, Shang J, Miyazaki K, Ohtsuka A, Katayama Y, Abe K. Dissociation and protection of the neurovascular unit after thrombolysis and reperfusion in ischemic rat brain. J Cereb Blood Flow Metab. 2009 Apr;29(4):715-25. doi: 10.1038/jcbfm.2008.164. Epub 2009 Jan 14. PMID: 19142198.
66. Yao RQ, Zhang L, Wang W, Li L. Cornel iridoid glycoside promotes neurogenesis and angiogenesis and improves neurological function after focal cerebral ischemia in rats. Brain Res Bull. 2009 Apr 6;79(1):69-76. doi: 10.1016/j.brainresbull.2008.12.010. Epub 2009 Jan 15. PMID: 19150488.
67. Atif F, Yousuf S, Agrawal SK. S-allyl L-cysteine diminishes cerebral ischemia-induced mitochondrial dysfunctions in hippocampus. Brain Res. 2009 Apr 10;1265:128-37. doi: 10.1016/j.brainres.2008.12.077. Epub 2009 Jan 15. PMID: 19401183.
68. Chang Y, Hsieh CY, Peng ZA, Yen TL, Hsiao G, Chou DS, Chen CM, Sheu JR. Neuroprotective mechanisms of puerarin in middle cerebral artery occlusion- induced brain infarction in rats. J Biomed Sci. 2009 Jan 19;16(1):9. doi: 10.1186/1423-0127-16-9. PMID: 19272172; PMCID: PMC2653511.
69. Li Y, Perry T, Kindy MS, Harvey BK, Tweedie D, Holloway HW, Powers K, Shen H, Egan JM, Sambamurti K, Brossi A, Lahiri DK, Mattson MP, Hoffer BJ, Wang Y, Greig NH. GLP-1 receptor stimulation preserves primary cortical and dopaminergic neurons in cellular and rodent models of stroke and Parkinsonism. Proc Natl Acad Sci U S A. 2009 Jan 27;106(4):1285-90. doi: 10.1073/pnas.0806720106. Epub 2009 Jan 21. PMID: 19164583; PMCID: PMC2633544.
70. Babu CS, Ramanathan M. Pre-ischemic treatment with memantine reversed the neurochemical and behavioural parameters but not energy metabolites in middle cerebral artery occluded rats. Pharmacol Biochem Behav. 2009 May;92(3):424-32. doi: 10.1016/j.pbb.2009.01.010. Epub 2009 Jan 23. PMID: 19463256.
71. Shih AY, Friedman B, Drew PJ, Tsai PS, Lyden PD, Kleinfeld D. Active dilation of penetrating arterioles restores red blood cell flux to penumbral neocortex after focal stroke. J Cereb Blood Flow Metab. 2009 Apr;29(4):738-51. doi: 10.1038/jcbfm.2008.166. Epub 2009 Jan 28. PMID: 19174826; PMCID: PMC2893883.
72. Reid MJ, Cross AK, Haddock G, Allan SM, Stock CJ, Woodroofe MN, Buttle DJ, Bunning RA. ADAMTS-9 expression is up-regulated following transient middle cerebral artery occlusion (tMCAo) in the rat. Neurosci Lett. 2009 Mar 20;452(3):252-7. doi: 10.1016/j.neulet.2009.01.058. Epub 2009 Jan 29. PMID: 19348733.
73. Sung JH, Cho EH, Kim MO, Koh PO. Identification of proteins differentially expressed by melatonin treatment in cerebral ischemic injury--a proteomics approach. J Pineal Res. 2009 Apr;46(3):300-6. doi: 10.1111/j.1600-079X.2008.00661.x. Epub 2009 Jan 31. PMID: 19196433.
74. Hall AA, Guyer AG, Leonardo CC, Ajmo CT Jr, Collier LA, Willing AE, Pennypacker KR. Human umbilical cord blood cells directly suppress ischemic oligodendrocyte cell death. J Neurosci Res. 2009 Feb;87(2):333-41. doi: 10.1002/jnr.21857. PMID: 18924174; PMCID: PMC2651744.
75. Kawaguchi AT, Kurita D, Furuya H, Yamano M, Ogata Y, Haida M. Liposome- encapsulated hemoglobin alleviates brain edema after permanent occlusion of the middle cerebral artery in rats. Artif Organs. 2009 Feb;33(2):153-8. doi: 10.1111/j.1525-1594.2008.00700.x. PMID: 19178460.
76. Keimpema E, Fokkens MR, Nagy Z, Agoston V, Luiten PG, Nyakas C, Boddeke HW, Copray JC. Early transient presence of implanted bone marrow stem cells reduces lesion size after cerebral ischaemia in adult rats. Neuropathol Appl Neurobiol. 2009 Feb;35(1):89-102. doi: 10.1111/j.1365-2990.2008.00961.x. PMID: 19187061.
77. Liu W, Hendren J, Qin XJ, Shen J, Liu KJ. Normobaric hyperoxia attenuates early blood-brain barrier disruption by inhibiting MMP-9-mediated occluding degradation in focal cerebral ischemia. J Neurochem. 2009 Feb;108(3):811-20. doi: 10.1111/j.1471-4159.2008.05821.x. PMID: 19187098; PMCID: PMC2676213.
78. Gautier S, Ouk T, Petrault O, Caron J, Bordet R. Neutrophils contribute to intracerebral haemorrhages after treatment with recombinant tissue plasminogen activator following cerebral ischaemia. Br J Pharmacol. 2009 Feb;156(4):673-9. doi: 10.1111/j.1476-5381.2009.00068.x. Epub 2009 Feb 4. PMID: 19210512; PMCID: PMC2697703.
79. Walberer M, Tschernatsch M, Fischer S, Ritschel N, Volk K, Friedrich C, Bachmann G, Mueller C, Kaps M, Nedelmann M, Blaes F, Preissner KT, Gerriets T. RNase therapy assessed by magnetic resonance imaging reduces cerebral edema and infarction size in acute stroke. Curr Neurovasc Res. 2009 Feb;6(1):12-9. doi: 10.2174/156720209787466037. PMID: 19355922.
80. Silasi G, MacLellan CL, Colbourne F. Use of telemetry blood pressure transmitters to measure intracranial pressure (ICP) in freely moving rats. Curr Neurovasc Res. 2009 Feb;6(1):62-9. doi: 10.2174/156720209787466046. PMID: 19355927.
81. Sayeed I, Parvez S, Wali B, Siemen D, Stein DG. Direct inhibition of the mitochondrial permeability transition pore: a possible mechanism for better neuroprotective effects of allopregnanolone over progesterone. Brain Res. 2009 Mar 31;1263:165-73. doi: 10.1016/j.brainres.2009.01.045. Epub 2009 Feb 3. PMID: 19368823.
82. Jia J, Hu YS, Wu Y, Liu G, Yu HX, Zheng QP, Zhu DN, Xia CM, Cao ZJ. Pre-ischemic treadmill training affects glutamate and gamma aminobutyric acid levels in the striatal dialysate of a rat model of cerebral ischemia. Life Sci. 2009 Apr 10;84(15-16):505-11. doi: 10.1016/j.lfs.2009.01.015. Epub 2009 Feb 4. PMID: 19302809.
83. Liu YP, Seçkin H, Izci Y, Du ZW, Yan YP, Başkaya MK. Neuroprotective effects of mesenchymal stem cells derived from human embryonic stem cells in transient focal cerebral ischemia in rats. J Cereb Blood Flow Metab. 2009 Apr;29(4):780-91. doi: 10.1038/jcbfm.2009.1. Epub 2009 Feb 11. PMID: 19209181.
84. Terao Y, Ohta H, Oda A, Nakagaito Y, Kiyota Y, Shintani Y. Macrophage inflammatory protein-3alpha plays a key role in the inflammatory cascade in rat focal cerebral ischemia. Neurosci Res. 2009 May;64(1):75-82. doi: 10.1016/j.neures.2009.01.017. Epub 2009 Feb 13. PMID: 19428685.
85. Tukhovskaya EA, Yukin AY, Khokhlova ON, Murashev AN, Vitek MP. COG1410, a novel apolipoprotein-E mimetic, improves functional and morphological recovery in a rat model of focal brain ischemia. J Neurosci Res. 2009 Feb 15;87(3):677-82. doi: 10.1002/jnr.21874. PMID: 18803296; PMCID: PMC2752425.
86. Liao W, Xie J, Zhong J, Liu Y, Du L, Zhou B, Xu J, Liu P, Yang S, Wang J, Han Z, Han ZC. Therapeutic effect of human umbilical cord multipotent mesenchymal stromal cells in a rat model of stroke. Transplantation. 2009 Feb 15;87(3):350-9. doi: 10.1097/TP.0b013e318195742e. PMID: 19202439.
87. Chen J, Cui X, Zacharek A, Ding GL, Shehadah A, Jiang Q, Lu M, Chopp M. Niaspan treatment increases tumor necrosis factor-alpha-converting enzyme and promotes arteriogenesis after stroke. J Cereb Blood Flow Metab. 2009 May;29(5):911-20. doi: 10.1038/jcbfm.2009.11. Epub 2009 Feb 18. PMID: 19223914; PMCID: PMC2782460.
88. Matsuda S, Umeda M, Uchida H, Kato H, Araki T. Alterations of oxidative stress markers and apoptosis markers in the striatum after transient focal cerebral ischemia in rats. J Neural Transm (Vienna). 2009 Apr;116(4):395-404. doi: 10.1007/s00702-009-0194-0. Epub 2009 Feb 24. PMID: 19238518.
89. Guerra-Crespo M, Gleason D, Sistos A, Toosky T, Solaroglu I, Zhang JH, Bryant PJ, Fallon JH. Transforming growth factor-alpha induces neurogenesis and behavioral improvement in a chronic stroke model. Neuroscience. 2009 May 5;160(2):470-83. doi: 10.1016/j.neuroscience.2009.02.029. Epub 2009 Feb 25. PMID: 19248822.
90. Lim CM, Kim SW, Park JY, Kim C, Yoon SH, Lee JK. Fluoxetine affords robust neuroprotection in the postischemic brain via its anti-inflammatory effect. J Neurosci Res. 2009 Mar;87(4):1037-45. doi: 10.1002/jnr.21899. PMID: 18855941.
91. Xu X, Ye L, Ruan Q. Environmental enrichment induces synaptic structural modification after transient focal cerebral ischemia in rats. Exp Biol Med (Maywood). 2009 Mar;234(3):296-305. doi: 10.3181/0804-RM-128. PMID: 19244205.
92. Vakili A, Hosseinzadeh SA, Khorasani MZ. Peripheral administration of carbenoxolone reduces ischemic reperfusion injury in transient model of cerebral ischemia. J Stroke Cerebrovasc Dis. 2009 Mar-Apr;18(2):81-5. doi: 10.1016/j.jstrokecerebrovasdis.2008.09.018. PMID: 19251182.
93. Solaroglu I, Cahill J, Tsubokawa T, Beskonakli E, Zhang JH. Granulocyte colony-stimulating factor protects the brain against experimental stroke via inhibition of apoptosis and inflammation. Neurol Res. 2009 Mar;31(2):167-72. doi: 10.1179/174313209X393582. PMID: 19298757.
94. Tang J, Li YJ, Mu J, Li Q, Yang DY, Xie P. Albumin ameliorates tissue plasminogen activator-mediated blood-brain barrier permeability and ischemic brain injury in rats. Neurol Res. 2009 Mar;31(2):189-94. doi: 10.1179/174313209X393898. PMID: 19298760.
95. Lu A, Kurosawa Y, Luskey K, Pyne-Geithman G, Caudell D, Clark J. Hemorrhagic profile of the fibrinolytic alfimeprase after ischemia and reperfusion. Neurol Res. 2009 Mar;31(2):209-14. doi: 10.1179/174313209X393933. PMID: 19298764.
96. Söderström I, Strand M, Ingridsson AC, Nasic S, Olsson T. 17beta-estradiol and enriched environment accelerate cognitive recovery after focal brain ischemia. Eur J Neurosci. 2009 Mar;29(6):1215-24. doi: 10.1111/j.1460-9568.2009.06662.x. PMID: 19302156.
97. Son HY, Han HS, Jung HW, Park YK. Panax notoginseng Attenuates the Infarct Volume in Rat Ischemic Brain and the Inflammatory Response of Microglia. J Pharmacol Sci. 2009 Mar;109(3):368-79. doi: 10.1254/jphs.08197fp. PMID: 19305121.
98. Cao S, Wang LC, Kwansa H, Roman RJ, Harder DR, Koehler RC. Endothelin rather than 20-HETE contributes to loss of pial arteriolar dilation during focal cerebral ischemia with and without polymeric hemoglobin transfusion. Am J Physiol Regul Integr Comp Physiol. 2009 May;296(5):R1412-8. doi: 10.1152/ajpregu.00003.2009. Epub 2009 Mar 4. PMID: 19261918; PMCID: PMC2689832.
99. Kozak A, Ergul A, El-Remessy AB, Johnson MH, Machado LS, Elewa HF, Abdelsaid M, Wiley DC, Fagan SC. Candesartan augments ischemia-induced proangiogenic state and results in sustained improvement after stroke. Stroke. 2009 May;40(5):1870-6. doi: 10.1161/STROKEAHA.108.537225. Epub 2009 Mar 5. PMID: 19265050; PMCID: PMC2716175.
100. Taskapilioglu MO, Alkan T, Goren B, Tureyen K, Sahin S, Taskapilioglu O, Korfali E. Neuronal protective effects of focal ischemic pre- and/or postconditioning on the model of transient focal cerebral ischemia in rats. J Clin Neurosci. 2009 May;16(5):693-7. doi: 10.1016/j.jocn.2008.07.077. Epub 2009 Mar 5. PMID: 19268597.

**Supplementary reference list for 2019**

This supplementary reference list includes all publications that were included in the analysis for the year 2019.

1. Altintas O, Antar V, Baran O, Karatas E, Altintas MO, Kesgin S, Buyukpinarbasili N, Kocyigit A, Asil T. Neuroprotective effects of hemicraniectomy in malignant middle cerebral artery infarctions: experimental study. J Neurosurg Sci. 2019 Dec;63(6):714-722. doi: 10.23736/S0390-5616.16.03444-5. Epub 2015 Oct 6. PMID: 26439453.
2. Nakamura H, Sasaki Y, Sasaki M, Kataoka-Sasaki Y, Oka S, Nakazaki M, Namioka T, Namioka A, Onodera R, Suzuki J, Nagahama H, Mikami T, Wanibuchi M, Kocsis JD, Honmou O. Elevated brain derived neurotrophic factor levels in plasma reflect in vivo functional viability of infused mesenchymal stem cells for stroke in rats. J Neurosurg Sci. 2019 Feb;63(1):42-49. doi: 10.23736/S0390-5616.17.03989-3. Epub 2017 Feb 8. PMID: 28181779.
3. Nasoohi S, Simani L, Khodagholi F, Nikseresht S, Faizi M, Naderi N. Coenzyme Q10 supplementation improves acute outcomes of stroke in rats pretreated with atorvastatin. Nutr Neurosci. 2019 Apr;22(4):264-272. doi: 10.1080/1028415X.2017.1376928. Epub 2017 Sep 26. PMID: 28946820.
4. Rutkai I, Merdzo I, Wunnava SV, Curtin GT, Katakam PV, Busija DW. Cerebrovascular function and mitochondrial bioenergetics after ischemia-reperfusion in male rats. J Cereb Blood Flow Metab. 2019 Jun;39(6):1056-1068. doi: 10.1177/0271678X17745028. Epub 2017 Dec 7. PMID: 29215305; PMCID: PMC6547195.
5. Poddar R, Rajagopal S, Winter L, Allan AM, Paul S. A peptide mimetic of tyrosine phosphatase STEP as a potential therapeutic agent for treatment of cerebral ischemic stroke. J Cereb Blood Flow Metab. 2019 Jun;39(6):1069-1084. doi: 10.1177/0271678X17747193. Epub 2017 Dec 7. PMID: 29215306; PMCID: PMC6547188.
6. Yang J, Qi J, Xiu B, Yang B, Niu C, Yang H. Reactive Oxygen Species Play a Biphasic Role in Brain Ischemia. J Invest Surg. 2019 Mar;32(2):97-102. doi: 10.1080/08941939.2017.1376131. Epub 2018 Feb 8. PMID: 29420085.
7. Chen B, Ng G, Gao Y, Low SW, Sandanaraj E, Ramasamy B, Sekar S, Bhakoo K, Soong TW, Nilius B, Tang C, Robins EG, Goggi J, Liao P. Non-Invasive Multimodality Imaging Directly Shows TRPM4 Inhibition Ameliorates Stroke Reperfusion Injury. Transl Stroke Res. 2019 Feb;10(1):91-103. doi: 10.1007/s12975-018-0621-3. Epub 2018 Mar 22. Erratum in: Transl Stroke Res. 2019 Apr 17;: PMID: 29569041; PMCID: PMC6327008.
8. Xie H, Yu K, Zhou N, Shen X, Tian S, Zhang B, Wang Y, Wu J, Liu G, Jiang C, Hu R, Ayata C, Wu Y. Enriched Environment Elicits Proangiogenic Mechanisms After Focal Cerebral Ischemia. Transl Stroke Res. 2019 Apr;10(2):150-159. doi: 10.1007/s12975-018-0629-8. Epub 2018 Apr 26. PMID: 29700717.
9. Ramírez-Sánchez J, Pires ENS, Meneghetti A, Hansel G, Nuñez-Figueredo Y, Pardo-Andreu GL, Ochoa-Rodríguez E, Verdecia-Reyes Y, Delgado-Hernández R, Salbego C, Souza DO. JM-20 Treatment After MCAO Reduced Astrocyte Reactivity and Neuronal Death on Peri-infarct Regions of the Rat Brain. Mol Neurobiol. 2019 Jan;56(1):502-512. doi: 10.1007/s12035-018-1087-8. Epub 2018 May 3. PMID: 29725905.
10. Yuen NY, Chechneva OV, Chen YJ, Tsai YC, Little LK, Dang J, Tancredi DJ, Conston J, Anderson SE, O'Donnell ME. Exacerbated brain edema in a rat streptozotocin model of hyperglycemic ischemic stroke: Evidence for involvement of blood-brain barrier Na-K-Cl cotransport and Na/H exchange. J Cereb Blood Flow Metab. 2019 Sep;39(9):1678-1692. doi: 10.1177/0271678X18770844. Epub 2018 May 9. PMID: 29739261; PMCID: PMC6727129.
11. Ding Y, Qian J, Li H, Shen H, Li X, Kong Y, Xu Z, Chen G. Effects of SC99 on cerebral ischemia-perfusion injury in rats: Selective modulation of microglia polarization to M2 phenotype via inhibiting JAK2-STAT3 pathway. Neurosci Res. 2019 May;142:58-68. doi: 10.1016/j.neures.2018.05.002. Epub 2018 May 12. PMID: 29763638.
12. Qin C, Zhou P, Wang L, Mamtilahun M, Li W, Zhang Z, Yang GY, Wang Y. Dl-3-N-butylphthalide attenuates ischemic reperfusion injury by improving the function of cerebral artery and circulation. J Cereb Blood Flow Metab. 2019 Oct;39(10):2011-2021. doi: 10.1177/0271678X18776833. Epub 2018 May 15. PMID: 29762050; PMCID: PMC6775578.
13. Abdul Y, Abdelsaid M, Li W, Webb RC, Sullivan JC, Dong G, Ergul A. Inhibition of Toll-Like Receptor-4 (TLR-4) Improves Neurobehavioral Outcomes After Acute Ischemic Stroke in Diabetic Rats: Possible Role of Vascular Endothelial TLR-4. Mol Neurobiol. 2019 Mar;56(3):1607-1617. doi: 10.1007/s12035-018-1184-8. Epub 2018 Jun 16. PMID: 29909454; PMCID: PMC6295357.
14. Jiang W, Hu W, Ye L, Tian Y, Zhao R, Du J, Shen B, Wang K. Contribution of Apelin-17 to Collateral Circulation Following Cerebral Ischemic Stroke. Transl Stroke Res. 2019 Jun;10(3):298-307. doi: 10.1007/s12975-018-0638-7. Epub 2018 Jun 18. PMID: 29916125.
15. Feng HX, Li CP, Shu SJ, Liu H, Zhang HY. A11, a novel diaryl acylhydrazone derivative, exerts neuroprotection against ischemic injury in vitro and in vivo. Acta Pharmacol Sin. 2019 Feb;40(2):160-169. doi: 10.1038/s41401-018-0028-4. Epub 2018 Jun 20. PMID: 29925921; PMCID: PMC6329839.
16. Shu L, Chen B, Chen B, Xu H, Wang G, Huang Y, Zhao Y, Gong H, Jiang M, Chen L, Liu X, Wang Y. Brain ischemic insult induces cofilin rod formation leading to synaptic dysfunction in neurons. J Cereb Blood Flow Metab. 2019 Nov;39(11):2181-2195. doi: 10.1177/0271678X18785567. Epub 2018 Jun 22. PMID: 29932353; PMCID: PMC6827117.
17. Morris-Blanco KC, Kim T, Bertogliat MJ, Mehta SL, Chokkalla AK, Vemuganti R. Inhibition of the Epigenetic Regulator REST Ameliorates Ischemic Brain Injury. Mol Neurobiol. 2019 Apr;56(4):2542-2550. doi: 10.1007/s12035-018-1254-y. Epub 2018 Jul 23. PMID: 30039336; PMCID: PMC6344325.
18. Nikseresht S, Khodagholi F, Ahmadiani A. Protective effects of ex-527 on cerebral ischemia-reperfusion injury through necroptosis signaling pathway attenuation. J Cell Physiol. 2019 Feb;234(2):1816-1826. doi: 10.1002/jcp.27055. Epub 2018 Aug 1. PMID: 30067864.
19. Jiang RH, Wu CJ, Xu XQ, Lu SS, Zu QQ, Zhao LB, Wang J, Liu S, Shi HB. Hypoxic conditioned medium derived from bone marrow mesenchymal stromal cells protects against ischemic stroke in rats. J Cell Physiol. 2019 Feb;234(2):1354-1368. doi: 10.1002/jcp.26931. Epub 2018 Aug 4. PMID: 30076722.
20. Gong P, Zhang Z, Zou C, Tian Q, Chen X, Hong M, Liu X, Chen Q, Xu Z, Li M, Wang J. Hippo/YAP signaling pathway mitigates blood-brain barrier disruption after cerebral ischemia/reperfusion injury. Behav Brain Res. 2019 Jan 1;356:8-17. doi: 10.1016/j.bbr.2018.08.003. Epub 2018 Aug 6. PMID: 30092249; PMCID: PMC6193462.
21. Chen X, Wu H, Chen H, Wang Q, Xie XJ, Shen J. Astragaloside VI Promotes Neural Stem Cell Proliferation and Enhances Neurological Function Recovery in Transient Cerebral Ischemic Injury via Activating EGFR/MAPK Signaling Cascades. Mol Neurobiol. 2019 Apr;56(4):3053-3067. doi: 10.1007/s12035-018-1294-3. Epub 2018 Aug 7. PMID: 30088176.
22. Andrews MMM, Peruzzaro S, Raupp S, Wilks J, Rossignol J, Dunbar GL. Using the behavioral flexibility operant task to detect long-term deficits in rats following middle cerebral artery occlusion. Behav Brain Res. 2019 Jan 1;356:1-7. doi: 10.1016/j.bbr.2018.08.008. Epub 2018 Aug 11. PMID: 30107224.
23. Lv H, Li J, Che YQ. MicroRNA-150 contributes to ischemic stroke through its effect on cerebral cortical neuron survival and function by inhibiting ERK1/2 axis via Mal. J Cell Physiol. 2019 Feb;234(2):1477-1490. doi: 10.1002/jcp.26960. Epub 2018 Aug 24. PMID: 30144062.
24. Zhou P, Du S, Zhou L, Sun Z, Zhuo LH, He G, Zhao Y, Wu Y, Zhang X. Tetramethylpyrazine‑2'O‑sodium ferulate provides neuroprotection against neuroinflammation and brain injury in MCAO/R rats by suppressing TLR-4/NF-κB signaling pathway. Pharmacol Biochem Behav. 2019 Jan;176:33-42. doi: 10.1016/j.pbb.2018.08.010. Epub 2018 Aug 29. PMID: 30171935.
25. Wang C, Wu C, Yan Z, Cheng X. Ameliorative effect of Xiaoyao-jieyu-san on post-stroke depression and its potential mechanisms. J Nat Med. 2019 Jan;73(1):76-84. doi: 10.1007/s11418-018-1243-5. Epub 2018 Sep 7. PMID: 30194657.
26. Han J, Feng Z, Xie Y, Li F, Lv B, Hua T, Zhang Z, Sun C, Su D, Ouyang Q, Cai Y, Zou Y, Tang Y, Sun H, Jiang X. Oncostatin M-induced upregulation of SDF-1 improves Bone marrow stromal cell migration in a rat middle cerebral artery occlusion stroke model. Exp Neurol. 2019 Mar;313:49-59. doi: 10.1016/j.expneurol.2018.09.005. Epub 2018 Sep 11. PMID: 30213507.
27. Chen ZZ, Gong X, Guo Q, Zhao H, Wang L. Bu Yang Huan Wu decoction prevents reperfusion injury following ischemic stroke in rats via inhibition of HIF-1 α, VEGF and promotion β-ENaC expression. J Ethnopharmacol. 2019 Jan 10;228:70-81. doi: 10.1016/j.jep.2018.09.017. Epub 2018 Sep 13. PMID: 30218809.
28. Li W, Valenzuela JP, Ward R, Abdelbary M, Dong G, Fagan SC, Ergul A. Post- stroke neovascularization and functional outcomes differ in diabetes depending on severity of injury and sex: Potential link to hemorrhagic transformation. Exp Neurol. 2019 Jan;311:106-114. doi: 10.1016/j.expneurol.2018.09.013. Epub 2018 Sep 19. PMID: 30243988; PMCID: PMC6585988.
29. Park J, Kim JH, Suk K, Han HS, Ohk B, Kim DG. Selective Brain Hypothermia Augmenting Neuroprotective Effects of Decompressive Craniectomy for Permanent Middle Cerebral Artery Infarction in a Rat Model. World Neurosurg. 2019 Jan;121:e181-e190. doi: 10.1016/j.wneu.2018.09.073. Epub 2018 Sep 25. PMID: 30261392.
30. Chu SF, Zhang Z, Zhou X, He WB, Chen C, Luo P, Liu DD, Ai QD, Gong HF, Wang ZZ, Sun HS, Feng ZP, Chen NH. Ginsenoside Rg1 protects against ischemic/reperfusion-induced neuronal injury through miR-144/Nrf2/ARE pathway. Acta Pharmacol Sin. 2019 Jan;40(1):13-25. doi: 10.1038/s41401-018-0154-z. Epub 2018 Sep 27. PMID: 30262824; PMCID: PMC6318278.
31. Bake S, Okoreeh A, Khosravian H, Sohrabji F. Insulin-like Growth Factor (IGF)-1 treatment stabilizes the microvascular cytoskeleton under ischemic conditions. Exp Neurol. 2019 Jan;311:162-172. doi: 10.1016/j.expneurol.2018.09.016. Epub 2018 Oct 1. PMID: 30287160; PMCID: PMC6263796.
32. Sheikh AM, Yano S, Mitaki S, Haque MA, Yamaguchi S, Nagai A. A Mesenchymal stem cell line (B10) increases angiogenesis in a rat MCAO model. Exp Neurol. 2019 Jan;311:182-193. doi: 10.1016/j.expneurol.2018.10.001. Epub 2018 Oct 3. PMID: 30291853.
33. Zhang J, Chen S, Shi W, Li M, Zhan Y, Yang L, Zou H, Lei J, Chai X, Gao K, Liu J, Wang W, Wang Y, Zhao H. Effects of *Xiaoshuan* Enteric-Coated Capsule on White and Gray Matter Injury Evaluated by Diffusion Tensor Imaging in Ischemic Stroke. Cell Transplant. 2019 Jun;28(6):671-683. doi: 10.1177/0963689718802755. Epub 2018 Oct 4. PMID: 30284459; PMCID: PMC6686435.
34. Qin Y, He Y, Zhu YM, Li M, Ni Y, Liu J, Zhang HL. CID1067700, a late endosome GTPase Rab7 receptor antagonist, attenuates brain atrophy, improves neurologic deficits and inhibits reactive astrogliosis in rat ischemic stroke. Acta Pharmacol Sin. 2019 Jun;40(6):724-736. doi: 10.1038/s41401-018-0166-8. Epub 2018 Oct 12. PMID: 30315251; PMCID: PMC6786391.
35. Liu Y, Xue X, Zhang H, Che X, Luo J, Wang P, Xu J, Xing Z, Yuan L, Liu Y, Fu X, Su D, Sun S, Zhang H, Wu C, Yang J. Neuronal-targeted TFEB rescues dysfunction of the autophagy-lysosomal pathway and alleviates ischemic injury in permanent cerebral ischemia. Autophagy. 2019 Mar;15(3):493-509. doi: 10.1080/15548627.2018.1531196. Epub 2018 Oct 18. PMID: 30304977; PMCID: PMC6351122.
36. Svoboda J, Litvinec A, Kala D, Pošusta A, Vávrová L, Jiruška P, Otáhal J. Strain differences in intraluminal thread model of middle cerebral artery occlusion in rats. Physiol Res. 2019 Mar 6;68(1):37-48. doi: 10.33549/physiolres.933958. Epub 2018 Oct 23. PMID: 30433803.
37. Huang R, Ma J, Niu B, Li J, Chang J, Zhang Y, Liu P, Luan X. MiR-34b Protects Against Focal Cerebral Ischemia-Reperfusion (I/R) Injury in Rat by Targeting Keap1. J Stroke Cerebrovasc Dis. 2019 Jan;28(1):1-9. doi: 10.1016/j.jstrokecerebrovasdis.2018.08.023. Epub 2018 Oct 24. PMID: 30539753.
38. Kong H, Zhang G, Cheng J, Shi R, Zhang M, Cao P, Zhao Y, Qu H, Wang Q. Distribution kinetics of puerarin in rat hippocampus after acute local cerebral ischemia. J Pharm Biomed Anal. 2019 Feb 5;164:196-201. doi: 10.1016/j.jpba.2018.10.038. Epub 2018 Oct 25. PMID: 30390562.
39. Yu H, Wang X, Kang F, Chen Z, Meng Y, Dai M. Neuroprotective effects of midazolam on focal cerebral ischemia in rats through anti‑apoptotic mechanisms. Int J Mol Med. 2019 Jan;43(1):443-451. doi: 10.3892/ijmm.2018.3973. Epub 2018 Nov 5. PMID: 30431057.
40. Yu H, Wang X, Kang F, Chen Z, Meng Y, Dai M. Propofol attenuates inflammatory damage on neurons following cerebral infarction by inhibiting excessive activation of microglia. Int J Mol Med. 2019 Jan;43(1):452-460. doi: 10.3892/ijmm.2018.3974. Epub 2018 Nov 5. PMID: 30431058.
41. Su X, Wu Z, Mai F, Fan Z, Du S, Qian H, Zhu J. 'Governor vessel-unblocking and mind-regulating' acupuncture therapy ameliorates cognitive dysfunction in a rat model of middle cerebral artery occlusion. Int J Mol Med. 2019 Jan;43(1):221-232. doi: 10.3892/ijmm.2018.3981. Epub 2018 Nov 6. PMID: 30431067; PMCID: PMC6257833.
42. Pravalika K, Sarmah D, Kaur H, Vats K, Saraf J, Wanve M, Kalia K, Borah A, Yavagal DR, Dave KR, Bhattacharya P. Trigonelline therapy confers neuroprotection by reduced glutathione mediated myeloperoxidase expression in animal model of ischemic stroke. Life Sci. 2019 Jan 1;216:49-58. doi: 10.1016/j.lfs.2018.11.014. Epub 2018 Nov 7. PMID: 30414429.
43. Cheng Q, Tong F, Shen Y, He C, Wang C, Ding F. Achyranthes bidentate polypeptide k improves long-term neurological outcomes through reducing downstream microvascular thrombosis in experimental ischemic stroke. Brain Res. 2019 Mar 1;1706:166-176. doi: 10.1016/j.brainres.2018.11.010. Epub 2018 Nov 8. PMID: 30414726.
44. Blixt FW, Haanes KA, Ohlsson L, Dreisig K, Fedulov V, Warfvinge K, Edvinsson L. MEK/ERK/1/2 sensitive vascular changes coincide with retinal functional deficit, following transient ophthalmic artery occlusion. Exp Eye Res. 2019 Feb;179:142-149. doi: 10.1016/j.exer.2018.11.003. Epub 2018 Nov 12. PMID: 30439349.
45. Zhang X, Zhao HH, Li D, Li HP. Neuroprotective effects of matrix metalloproteinases in cerebral ischemic rats by promoting activation and migration of astrocytes and microglia. Brain Res Bull. 2019 Mar;146:136-142. doi: 10.1016/j.brainresbull.2018.11.003. Epub 2018 Nov 13. PMID: 30445183.
46. Zuo W, Liu Z, Yan F, Mei D, Hu X, Zhang B. Hyperglycemia abolished Drp-1-mediated mitophagy at the early stage of cerebral ischemia. Eur J Pharmacol. 2019 Jan 15;843:34-44. doi: 10.1016/j.ejphar.2018.11.011. Epub 2018 Nov 14. PMID: 30447185.
47. He H, Zeng Q, Huang G, Lin Y, Lin H, Liu W, Lu P. Bone marrow mesenchymal stem cell transplantation exerts neuroprotective effects following cerebral ischemia/reperfusion injury by inhibiting autophagy via the PI3K/Akt pathway. Brain Res. 2019 Mar 15;1707:124-132. doi: 10.1016/j.brainres.2018.11.018. Epub 2018 Nov 16. PMID: 30448444.
48. Feczkó T, Piiper A, Ansar S, Blixt FW, Ashtikar M, Schiffmann S, Ulshöfer T, Parnham MJ, Harel Y, Israel LL, Lellouche JP, Wacker MG. Stimulating brain recovery after stroke using theranostic albumin nanocarriers loaded with nerve growth factor in combination therapy. J Control Release. 2019 Jan 10;293:63-72. doi: 10.1016/j.jconrel.2018.11.017. Epub 2018 Nov 17. PMID: 30458203.
49. Braun T, Pukropski J, Yeniguen M, El-Shazly J, Schoenburg M, Gerriets T, Kaps M, Tschernatsch M, Juenemann M. Inter- and intra-rater reliability of computer-assisted planimetry in experimental stroke research. J Neurosci Methods. 2019 Jan 15;312:12-15. doi: 10.1016/j.jneumeth.2018.11.012. Epub 2018 Nov 19. PMID: 30465797.
50. Lyden PD, Lamb J, Kothari S, Toossi S, Boitano P, Rajput PS. Differential effects of hypothermia on neurovascular unit determine protective or toxic results: Toward optimized therapeutic hypothermia. J Cereb Blood Flow Metab. 2019 Sep;39(9):1693-1709. doi: 10.1177/0271678X18814614. Epub 2018 Nov 21. PMID: 30461327; PMCID: PMC6727141.
51. Hu J, Li C, Hua Y, Zhang B, Gao BY, Liu PL, Sun LM, Lu RR, Wang YY, Bai YL. Constrained-induced movement therapy promotes motor function recovery by enhancing the remodeling of ipsilesional corticospinal tract in rats after stroke. Brain Res. 2019 Apr 1;1708:27-35. doi: 10.1016/j.brainres.2018.11.011. Epub 2018 Nov 22. PMID: 30471245.
52. Ma J, Bao L, Xia X, Feng Q, Zhou Y, Wang Y, Cao Z. miR-128b Promotes Cerebral Infarction by Regulating the Expressions of BCL-2 and CAPASE3. World Neurosurg. 2019 Mar;123:e245-e251. doi: 10.1016/j.wneu.2018.11.144. Epub 2018 Nov 24. PMID: 30481625.
53. Otsuka S, Sakakima H, Terashi T, Takada S, Nakanishi K, Kikuchi K. Preconditioning exercise reduces brain damage and neuronal apoptosis through enhanced endogenous 14-3-3γ after focal brain ischemia in rats. Brain Struct Funct. 2019 Mar;224(2):727-738. doi: 10.1007/s00429-018-1800-4. Epub 2018 Nov 26. Erratum in: Brain Struct Funct. 2018 Dec 17;: PMID: 30478609.
54. Yu J, Moon J, Jang J, Choi JI, Jung J, Hwang S, Kim M. Reliability of behavioral tests in the middle cerebral artery occlusion model of rat. Lab Anim. 2019 Oct;53(5):478-490. doi: 10.1177/0023677218815210. Epub 2018 Nov 27. PMID: 30482088.
55. Rajan WD, Wojtas B, Gielniewski B, Gieryng A, Zawadzka M, Kaminska B. Dissecting functional phenotypes of microglia and macrophages in the rat brain after transient cerebral ischemia. Glia. 2019 Feb;67(2):232-245. doi: 10.1002/glia.23536. Epub 2018 Nov 28. PMID: 30485549.
56. Sayeed I, Turan N, Stein DG, Wali B. Vitamin D deficiency increases blood- brain barrier dysfunction after ischemic stroke in male rats. Exp Neurol. 2019 Feb;312:63-71. doi: 10.1016/j.expneurol.2018.11.005. Epub 2018 Nov 28. PMID: 30502340.
57. Li Z, Yulei J, Yaqing J, Jinmin Z, Xinyong L, Jing G, Min L. Protective effects of tetramethylpyrazine analogue Z-11 on cerebral ischemia reperfusion injury. Eur J Pharmacol. 2019 Feb 5;844:156-164. doi: 10.1016/j.ejphar.2018.11.031. Epub 2018 Nov 29. PMID: 30502344.
58. Li Q, Tian Z, Wang M, Kou J, Wang C, Rong X, Li J, Xie X, Pang X. Luteoloside attenuates neuroinflammation in focal cerebral ischemia in rats via regulation of the PPARγ/Nrf2/NF-κB signaling pathway. Int Immunopharmacol. 2019 Jan;66:309-316. doi: 10.1016/j.intimp.2018.11.044. Epub 2018 Nov 29. PMID: 30502652.
59. Luo L, Li C, Deng Y, Wang Y, Meng P, Wang Q. High-Intensity Interval Training on Neuroplasticity, Balance between Brain-Derived Neurotrophic Factor and Precursor Brain-Derived Neurotrophic Factor in Poststroke Depression Rats. J Stroke Cerebrovasc Dis. 2019 Mar;28(3):672-682. doi: 10.1016/j.jstrokecerebrovasdis.2018.11.009. Epub 2018 Nov 30. PMID: 30503681.
60. Peng L, Zhao Y, Li Y, Zhou Y, Li L, Lei S, Yu S, Zhao Y. Effect of DJ-1 on the neuroprotection of astrocytes subjected to cerebral ischemia/reperfusion injury. J Mol Med (Berl). 2019 Feb;97(2):189-199. doi: 10.1007/s00109-018-1719-5. Epub 2018 Nov 30. PMID: 30506316; PMCID: PMC6348070.
61. Xu K, Lee JY, Kaneko Y, Tuazon JP, Vale F, van Loveren H, Borlongan CV. Human stem cells transplanted into the rat stroke brain migrate to the spleen via lymphatic and inflammation pathways. Haematologica. 2019 May;104(5):1062-1073. doi: 10.3324/haematol.2018.206581. Epub 2018 Dec 4. PMID: 30514806; PMCID: PMC6518907.
62. Peng JJ, Sha R, Li MX, Chen LT, Han XH, Guo F, Chen H, Huang XL. Repetitive transcranial magnetic stimulation promotes functional recovery and differentiation of human neural stem cells in rats after ischemic stroke. Exp Neurol. 2019 Mar;313:1-9. doi: 10.1016/j.expneurol.2018.12.002. Epub 2018 Dec 4. PMID: 30529277.
63. Zhang G, Ge M, Han Z, Wang S, Yin J, Peng L, Xu F, Zhang Q, Dai Z, Xie L, Li Y, Si J, Ma K. Wnt/β-catenin signaling pathway contributes to isoflurane postconditioning against cerebral ischemia-reperfusion injury and is possibly related to the transforming growth factorβ1/Smad3 signaling pathway. Biomed Pharmacother. 2019 Feb;110:420-430. doi: 10.1016/j.biopha.2018.11.143. Epub 2018 Dec 5. PMID: 30530044.
64. Hua S, Wang B, Chen R, Zhang Y, Zhang Y, Li T, Dong L, Fu X. Neuroprotective Effect of Dichloromethane Extraction From Piper nigrum L. and Piper longum L. on Permanent Focal Cerebral Ischemia Injury in Rats. J Stroke Cerebrovasc Dis. 2019 Mar;28(3):751-760. doi: 10.1016/j.jstrokecerebrovasdis.2018.11.018. Epub 2018 Dec 8. PMID: 30528673.
65. Qian J, Wang L, Li Q, Sha D, Wang J, Zhang J, Xu P, Fan G. Ultrasound- targeted microbubble enhances migration and therapeutic efficacy of marrow mesenchymal stem cell on rat middle cerebral artery occlusion stroke model. J Cell Biochem. 2019 Mar;120(3):3315-3322. doi: 10.1002/jcb.27600. Epub 2018 Dec 9. PMID: 30537289.
66. Cen J, Zhao N, Huang WW, Liu L, Xie YY, Gan Y, Wang CJ, Ji BS. Polyamine analogue QMA attenuated ischemic injury in MCAO rats via ERK and Akt activated Nrf2/HO-1 signaling pathway. Eur J Pharmacol. 2019 Feb 5;844:165-174. doi: 10.1016/j.ejphar.2018.12.015. Epub 2018 Dec 11. PMID: 30550742.
67. Cheng CY, Kao ST, Lee YC. Ferulic acid ameliorates cerebral infarction by activating Akt/mTOR/4E‑BP1/Bcl‑2 anti‑apoptotic signaling in the penumbral cortex following permanent cerebral ischemia in rats. Mol Med Rep. 2019 Feb;19(2):792-804. doi: 10.3892/mmr.2018.9737. Epub 2018 Dec 11. PMID: 30569126.
68. Xie G, Song C, Lin X, Yang M, Fan X, Liu W, Tao J, Chen L, Huang J. Electroacupuncture Regulates Hippocampal Synaptic Plasticity via Inhibiting Janus-Activated Kinase 2/Signal Transducer and Activator of Transcription 3 Signaling in Cerebral Ischemic Rats. J Stroke Cerebrovasc Dis. 2019 Mar;28(3):792-799. doi: 10.1016/j.jstrokecerebrovasdis.2018.11.025. Epub 2018 Dec 12. PMID: 30552029.
69. Li F, Yang B, Li T, Gong X, Zhou F, Hu Z. HSPB8 over-expression prevents disruption of blood-brain barrier by promoting autophagic flux after cerebral ischemia/reperfusion injury. J Neurochem. 2019 Jan;148(1):97-113. doi: 10.1111/jnc.14626. Epub 2018 Dec 13. PMID: 30422312.
70. Xie Q, Cheng J, Pan G, Wu S, Hu Q, Jiang H, Wang Y, Xiong J, Pang Q, Chen X. Treadmill exercise ameliorates focal cerebral ischemia/reperfusion-induced neurological deficit by promoting dendritic modification and synaptic plasticity via upregulating caveolin-1/VEGF signaling pathways. Exp Neurol. 2019 Mar;313:60-78. doi: 10.1016/j.expneurol.2018.12.005. Epub 2018 Dec 13. PMID: 30552877.
71. Zhong Y, Yu C, Qin W. LncRNA SNHG14 promotes inflammatory response induced by cerebral ischemia/reperfusion injury through regulating miR-136-5p /ROCK1. Cancer Gene Ther. 2019 Jul;26(7-8):234-247. doi: 10.1038/s41417-018-0067-5. Epub 2018 Dec 14. PMID: 30546117; PMCID: PMC6760557.
72. Wu C, Chen J, Yang R, Duan F, Li S, Chen X. Mitochondrial protective effect of neferine through the modulation of nuclear factor erythroid 2-related factor 2 signalling in ischaemic stroke. Br J Pharmacol. 2019 Feb;176(3):400-415. doi: 10.1111/bph.14537. Epub 2018 Dec 18. PMID: 30414381; PMCID: PMC6329622.
73. Yang D, Ma L, Wang P, Yang D, Zhang Y, Zhao X, Lv J, Zhang J, Zhang Z, Gao F. Normobaric oxygen inhibits AQP4 and NHE1 expression in experimental focal ischemic stroke. Int J Mol Med. 2019 Mar;43(3):1193-1202. doi: 10.3892/ijmm.2018.4037. Epub 2018 Dec 20. PMID: 30592266; PMCID: PMC6365048.
74. Li C, Zhang B, Tian S, Hu J, Gao B, Liu P, Hua Y, Bao W, Guan Y, Bai Y. Early wheel-running promotes functional recovery by improving mitochondria metabolism in olfactory ensheathing cells after ischemic stroke in rats. Behav Brain Res. 2019 Apr 1;361:32-38. doi: 10.1016/j.bbr.2018.12.038. Epub 2018 Dec 21. PMID: 30583029.
75. Wu D, Zhi X, Duan Y, Zhang M, An H, Wei W, Dong K, Zhang Y, Shi J, He X, Zhang J, Wu C, Meng R, Ding Y, Ji X. Inflammatory cytokines are involved in dihydrocapsaicin (DHC) and regional cooling infusion (RCI)-induced neuroprotection in ischemic rat. Brain Res. 2019 May 1;1710:173-180. doi: 10.1016/j.brainres.2018.12.033. Epub 2018 Dec 22. PMID: 30584925.
76. Wen L, Liu L, Li J, Tong L, Zhang K, Zhang Q, Li C. NDRG4 protects against cerebral ischemia injury by inhibiting p53-mediated apoptosis. Brain Res Bull. 2019 Mar;146:104-111. doi: 10.1016/j.brainresbull.2018.12.010. Epub 2018 Dec 26. PMID: 30593880.
77. Duan W, Wang L, Lv J, Gao K, Lu Y, Qin S, Ma X, Li J, Ge X. Metabolomics Study on the Effects of Salvianolic Acid B and Borneol for Treating Cerebral Ischemia in Rats by Ultra-Performance Liquid Chromatography Quadrupole Time-of- Flight Mass Spectrometry. Rejuvenation Res. 2019 Aug;22(4):313-324. doi: 10.1089/rej.2018.2099. Epub 2018 Dec 28. PMID: 30411995.
78. Zhao G, Yang L, Wang S, Cai M, Sun S, Dong H, Xiong L. TREK-2 Mediates the Neuroprotective Effect of Isoflurane Preconditioning Against Acute Cerebral Ischemia in the Rat. Rejuvenation Res. 2019 Aug;22(4):325-334. doi: 10.1089/rej.2017.2039. Epub 2018 Dec 28. PMID: 30412001.
79. Yao X, Yao R, Yi J, Huang F. Upregulation of miR-496 decreases cerebral ischemia/reperfusion injury by negatively regulating BCL2L14. Neurosci Lett. 2019 Mar 23;696:197-205. doi: 10.1016/j.neulet.2018.12.039. Epub 2018 Dec 28. PMID: 30597231.
80. Cheng CY, Kao ST, Lee YC. Ferulic Acid Exerts Anti-apoptotic Effects against Ischemic Injury by Activating HSP70/Bcl-2- and HSP70/Autophagy-Mediated Signaling after Permanent Focal Cerebral Ischemia in Rats. Am J Chin Med. 2019;47(1):39-61. doi: 10.1142/S0192415X19500034. Epub 2019 Jan 7. PMID: 30612456.
81. Bian KY, Jin HF, Sun W, Sun YJ. DCA can improve the ACI-induced neurological impairment through negative regulation of Nrf2 signaling pathway. Eur Rev Med Pharmacol Sci. 2019 Jan;23(1):343-351. doi: 10.26355/eurrev_201901_16782. PMID: 30657576.
82. Wang BX, Xu JJ, Hu J, Hu ML, Huang JM, Zhu XD. Effects of miR-153 on angiogenesis in MCAO rats through Shh signaling pathway. Eur Rev Med Pharmacol Sci. 2019 Jan;23(2):732-739. doi: 10.26355/eurrev_201901_16887. PMID: 30720181.
83. Zhang DL, Liu X, Wang Q, Li N, Wu SH, Wang C. Downregulation of microRNA-196a attenuates ischemic brain injury in rats by directly targeting HMGA1. Eur Rev Med Pharmacol Sci. 2019 Jan;23(2):740-748. doi: 10.26355/eurrev_201901_16888. PMID: 30720182.
84. Qin A, Zhang Q, Wang J, Sayeed I, Stein DG. Is a combination of progesterone and chloroquine more effective than either alone in the treatment of cerebral ischemic injury? Restor Neurol Neurosci. 2019;37(1):1-10. doi: 10.3233/RNN-180837. PMID: 30741704.
85. Lu W, Xv L, Wen J. Protective effect of extract of the Camellia japonica L. on cerebral ischemia-reperfusion injury in rats. Arq Neuropsiquiatr. 2019 Jan;77(1):39-46. doi: 10.1590/0004-282X20180146. PMID: 30758441.
86. Zhang L, Li S, Chen L, Li J, Zhang Z, Yang Y, Wang X, Liu J. Cerebellar fastigial nucleus electrical stimulatin protects against cerebral ischemic damage by upregulating telomerase activity. Restor Neurol Neurosci. 2019;37(2):131-141. doi: 10.3233/RNN-180876. PMID: 30988241.
87. Xu B, He X, Sui Y, Wang X, Wang X, Ren L, Zhai YX. Ginkgetin aglycone attenuates neuroinflammation and neuronal injury in the rats with ischemic stroke by modulating STAT3/JAK2/SIRT1. Folia Neuropathol. 2019;57(1):16-23. doi: 10.5114/fn.2019.83827. PMID: 31038184.
88. Orgah JO, Ren J, Liu X, Orgah EA, Gao XM, Zhu Y. Danhong injection facilitates recovery of post-stroke motion deficit via Parkin-enhanced mitochondrial function. Restor Neurol Neurosci. 2019;37(4):375-395. doi: 10.3233/RNN-180828. PMID: 31282440.
89. Ben RJ, Jao JC, Chang CY, Tzeng JS, Hwang LC, Chen PC. Longitudinal investigation of ischemic stroke using magnetic resonance imaging: Animal model. J Xray Sci Technol. 2019;27(5):935-947. doi: 10.3233/XST-190538. PMID: 31306147.
90. Li S, Chen L, Zhou X, Li J, Liu J. miRNA-223-3p and let-7b-3p as potential blood biomarkers associated with the ischemic penumbra in rats. Acta Neurobiol Exp (Wars). 2019;79(2):205-216. PMID: 31342956.
91. Wang Y, Wu Y, Liang C, Tan R, Tan L, Tan R. Pharmacodynamic Effect of Ellagic Acid on Ameliorating Cerebral Ischemia/Reperfusion Injury. Pharmacology. 2019;104(5-6):320-331. doi: 10.1159/000502401. Epub 2019 Aug 30. PMID: 31473749.
92. Xu H, Nie B, Liu L, Zhang C, Zhang Z, Xu M, Mei Y. Curcumin Prevents Brain Damage and Cognitive Dysfunction During Ischemic-reperfusion Through the Regulation of miR-7-5p. Curr Neurovasc Res. 2019;16(5):441-454. doi: 10.2174/1567202616666191029113633. PMID: 31660818.
93. Song H, Zhang X, Chen R, Miao J, Wang L, Cui L, Ji H, Liu Y. Cortical Neuron-Derived Exosomal MicroRNA-181c-3p Inhibits Neuroinflammation by Downregulating CXCL1 in Astrocytes of a Rat Model with Ischemic Brain Injury. Neuroimmunomodulation. 2019;26(5):217-233. doi: 10.1159/000502694. Epub 2019 Oct 30. PMID: 31665717.
94. Wu P, Yan XS, Zhou LL, Liu XL, Huo DS, Song W, Fang X, Wang H, Yang ZJ, Jia JX. Involvement of apoptosis in the protective effects of Dracocephalum moldavaica in cerebral ischemia reperfusion rat model. J Toxicol Environ Health A. 2019;82(19):1036-1044. doi: 10.1080/15287394.2019.1684707. Epub 2019 Nov 18. PMID: 31736438.
95. Zhang D, Qian J, Zhang P, Li H, Shen H, Li X, Chen G. Gasdermin D serves as a key executioner of pyroptosis in experimental cerebral ischemia and reperfusion model both in vivo and in vitro. J Neurosci Res. 2019 Jun;97(6):645-660. doi: 10.1002/jnr.24385. Epub 2019 Jan 2. PMID: 30600840.
96. Ruan W, Li J, Xu Y, Wang Y, Zhao F, Yang X, Jiang H, Zhang L, Saavedra JM, Shi L, Pang T. MALAT1 Up-Regulator Polydatin Protects Brain Microvascular Integrity and Ameliorates Stroke Through C/EBPβ/MALAT1/CREB/PGC-1α/PPARγ Pathway. Cell Mol Neurobiol. 2019 Mar;39(2):265-286. doi: 10.1007/s10571-018-00646-4. Epub 2019 Jan 3. PMID: 30607811.
97. Liu K, Guo L, Zhou Z, Pan M, Yan C. Mesenchymal stem cells transfer mitochondria into cerebral microvasculature and promote recovery from ischemic stroke. Microvasc Res. 2019 May;123:74-80. doi: 10.1016/j.mvr.2019.01.001. Epub 2019 Jan 3. PMID: 30611747.
98. Hu J, Shen W. microRNA-196a attenuates ischemic brain injury in rats by directly targeting high mobility group A1. Exp Ther Med. 2019 Mar;17(3):1579-1586. doi: 10.3892/etm.2019.7152. Epub 2019 Jan 4. PMID: 30783424; PMCID: PMC6364231.
99. van Kralingen JC, McFall A, Ord ENJ, Coyle TF, Bissett M, McClure JD, McCabe C, Macrae IM, Dawson J, Work LM. Altered Extracellular Vesicle MicroRNA Expression in Ischemic Stroke and Small Vessel Disease. Transl Stroke Res. 2019 Oct;10(5):495-508. doi: 10.1007/s12975-018-0682-3. Epub 2019 Jan 7. PMID: 30617992; PMCID: PMC6733813.
100. Hu S, Cheng D, Peng D, Tan J, Huang Y, Chen C. Leptin attenuates cerebral ischemic injury in rats by modulating the mitochondrial electron transport chain via the mitochondrial STAT3 pathway. Brain Behav. 2019 Feb;9(2):e01200. doi: 10.1002/brb3.1200. Epub 2019 Jan 10. PMID: 30632310; PMCID: PMC6379515.
